# Epithelial−mesenchymal transition induced by tumor cell-intrinsic PD-L1 signaling predicts a poor response to immune checkpoint inhibitors in PD-L1-high lung cancer

**DOI:** 10.1038/s41416-024-02698-4

**Published:** 2024-05-10

**Authors:** Hyein Jeong, Jaemoon Koh, Sehui Kim, Seung Geun Song, Soo Hyun Lee, Youngjoo Jeon, Chul-Hwan Lee, Bhumsuk Keam, Se-Hoon Lee, Doo Hyun Chung, Yoon Kyung Jeon

**Affiliations:** 1 Cancer Research Institute, Seoul National University, Seoul, Republic of Korea; 2 Interdiscipilinary Program of Cancer Biology, Seoul National University Graduate School, Seoul, Republic of Korea; 3 Department of Pathology, Seoul National University Hospital, Seoul National University College of Medicine, Seoul, Republic of Korea; 4 Department of Pathology, Korea University Guro Hospital, Korea University College of Medicine, Seoul, Republic of Korea; 5 Department of Biomedical Sciences, Seoul National University College of Medicine, Seoul, Republic of Korea; 6 Department of Pharmacology, Seoul National University College of Medicine, Seoul, Republic of Korea; 7 BK21 FOUR Biomedical Science Project, Seoul National University College of Medicine, Seoul, Republic of Korea; 8 Department of Internal Medicine, Seoul National University Hospital, Seoul National University College of Medicine, Seoul, Republic of Korea; 9 Division of Hematology-Oncology, Department of Medicine, Samsung Medical Center, Sungkyunkwan University School of Medicine, Seoul, Republic of Korea; 10 Department of Health Sciences and Technology, Samsung Advanced Institute of Health Sciences and Technology, Sungkyunkwan University, Seoul, Republic of Korea

**Keywords:** Non-small-cell lung cancer, Cancer microenvironment, Cancer immunotherapy, Predictive markers

## Abstract

**Background:**

We investigated the role of tumor cell-intrinsic PD-L1 signaling in the epithelial−mesenchymal transition (EMT) in non-small-cell lung cancer (NSCLC) and the role of EMT as a predictive biomarker for immune checkpoint inhibitor (ICI) therapy.

**Methods:**

PD-L1-overexpressing or PD-L1-knockdown NSCLC cells underwent RNA-seq and EMT phenotype assessment. Mouse lung cancer LLC cells were injected into nude mice. Two cohorts of patients with NSCLC undergoing ICI therapy were analyzed.

**Results:**

RNA-seq showed that EMT pathways were enriched in PD-L1-high NSCLC cells. EMT was enhanced by PD-L1 in NSCLC cells, which was mediated by transforming growth factor-β (TGFβ). PD-L1 promoted the activation of p38-MAPK by binding to and inhibiting the protein phosphatase PPM1B, thereby increasing the TGFβ production. Tumor growth and metastasis increased in nude mice injected with PD-L1-overexpressing LLC cells. In the ICI cohort, EMT signature was higher in patients with progressive disease than in those with responses, and EMT was significantly associated with poor survival in PD-L1-high NSCLC. In PD-L1-high NSCLC, EMT was associated with increased M2-macrophage and regulatory T-cell infiltrations and decreased cytotoxic T-cell infiltration.

**Conclusions:**

Tumor cell-intrinsic PD-L1 function contributes to NSCLC progression by promoting EMT. EMT may predict an unfavorable outcome after ICI therapy in PD-L1-high NSCLC.

## Background

The immune checkpoint molecule programmed death ligand-1 (PD-L1) suppresses immunity when PD-L1 on the cell surface is engaged by programmed death-1 (PD-1) on immune cells [1]. Cancer immunotherapy based on blockade of the PD-1/PD-L1 interaction by immune checkpoint inhibitors (ICIs) has been established as one of the main therapeutic strategies for cancer, particularly non-small-cell lung cancer (NSCLC) [2–5]. However, the response rate to PD-1/PD-L1 blockade is approximately 30% in patients with NSCLC, and several biomarkers have been demonstrated to predict a response to ICI [6]. Among these biomarkers, PD-L1 expression is the most widely used practical biomarker for PD-1/PD-L1 blockade [7]. However, the response rate to PD-1/PD-L1 blockade is approximately 40% even in patients with PD-L1-positive NSCLC [8]. Thus, a biomarker for ICI therapy in patients with PD-L1-positive NSCLC remains a clinically unmet need.

The role of PD-L1 expression as an immunotherapy biomarker might be complicated by the cell-intrinsic function of PD-L1. Although most researchers have focused on the conventional cell-extrinsic role of PD-L1 as an immune checkpoint ligand, the tumor cell-intrinsic signals of PD-L1 were recently revisited and are being increasingly reported to exhibit variable functions [9, 10]. PD-L1 intrinsic function involves cancer cell survival, invasion, stemness, glycolysis, chemotherapy resistance, DNA damage response, and interferon response pathways [9–15]. Moreover, PD-L1 intrinsic function was associated with resistance to anti-PD-1 therapy in murine melanoma and colon cancer models [16, 17]. However, the tumor cell-intrinsic role of PD-L1 in cancer biology and therapeutic development has not been fully elucidated, and its mechanism remains unclear.

The epithelial−mesenchymal transition (EMT) plays an important role in cancer progression and metastasis [18–20]. A positive correlation between PD-L1 expression and the EMT phenotype has been reported in NSCLC, breast cancer, head-neck squamous cell carcinoma, and other tumors [21–25]. These observations suggest that crosstalk exists between EMT and PD-L1. ZEB1, an EMT transcription factor, increases PD-L1 expression by suppressing miR-200 and subsequently alleviating miR-200-mediated PD-L1 downregulation [26]. By contrast, tumor cell-intrinsic PD-L1 contributes to the EMT phenotype and tumor progression in melanoma, ovarian cancer, breast cancer, nasopharyngeal carcinoma, and lung cancer [12, 14, 27, 28]. In a murine melanoma model, tumor cell-intrinsic PD-L1 and transforming growth factor-β (TGFβ) upregulated each other in a bidirectional way and involved in EMT [29]. However, in vivo evidence for the tumor-promoting function of tumor cell-intrinsic PD-L1 using a lung cancer model is limited, and the mechanism of PD-L1-induced EMT in NSCLC remains unclear.

EMT is a common phenomenon during or after therapy, and therapy-induced EMT implies therapeutic resistance and cancer progression [18–20]. However, the association between EMT and immunotherapy remains to be elucidated. Immunosuppression by EMT was recently demonstrated to be a mechanism that accelerates cancer metastasis primarily using melanoma and breast cancer models [30–33]. Tumors with EMT signatures are characterized by immune activation signatures including increased expression of both immune checkpoint molecules and immune stimulatory molecules as well as high infiltration of both immune effector and immune suppressive cells [24, 25, 34]. These findings suggest that EMT might contribute to immunosuppression in patients with NSCLC despite the inflamed tumor microenvironment (TME) and that EMT is probably related to the responsiveness to ICI. However, only a few small cohort studies have provided data regarding the implication of EMT in the responsiveness to ICI among patients with NSCLC [34, 35], and the relationship between intrinsic PD-L1 function, EMT, and ICI response has never been addressed.

Therefore, the present study investigated the role and mechanism of cell-intrinsic PD-L1 signaling in EMT and tumor progression of NSCLC in vitro and in vivo and explored the role of PD-L1-induced EMT as a predictive biomarker for ICI therapy in patients with NSCLC, particularly in the context of PD-L1 expression.

## Methods

### Cell lines and reagents

The human NSCLC cell line A549, human embryonic kidney cell line Lenti-X-293T, and HEK293T cells were cultured in DMEM (Biowest, Nuaillé, France) supplemented with 10% fetal bovine serum (FBS) and 100 units/mL penicillin/streptomycin. The human NSCLC lines H460, H596, H522, H820, H1975, HCC827, H358, H3122, H1650, H1703, EBC-1, Calu-1, and HCC15 were cultured in RPMI 1640 (Biowest) supplemented with 10% FBS and 100 units/mL penicillin/streptomycin. The murine Lewis lung carcinoma cell line LLC was cultured in complete RPMI medium. All cell lines were purchased from American Type Culture Collection (Manassas, VA, USA) or Korean Cell Line Bank (Seoul, Republic of Korea).

Recombinant human PD-1 (rhPD-1) (1086-PD), recombinant human interferon-γ (IFNγ) (285-IF-100/CF), and anti-TGFβ neutralizing antibodies (MAB1835-SP) were purchased from R&D Systems (Minneapolis, MN, USA). P38 MAPK inhibitor, SB203580 (S8307), was purchased from Sigma-Aldrich (Burlington, MA, USA).

### Expression vectors, short hairpin RNAs, and small interfering RNAs

Human PD-L1-expressing plasmids (HG10084-UT), PD-L1-N-Flag tagged plasmids (HG10084-NF), and PPM1B-Myc tagged expression vector (HG12224-CM) were purchased from Sino Biological (Beijing, China). Small interfering RNAs (siRNAs) for human PD-L1 (NM 014143.2) knockdown and human PPM1B (NM 002706.5) knockdown were designed and synthesized by Bioneer (Daejeon, Republic of Korea). The sequences of human PD-L1 siRNAs were as follows: sense 5′-CUG AGA AUC AAC ACA ACA A (dTdT)-3′ and antisense 5′-UUG UUG UGU UGA UUC UCA G (dTdT)-3′. The sequences of human PPM1B siRNAs were as follows: sense 5′-CAG AGU UGG AUA AGC ACU U (dTdT)-3′ and antisense 5′-AAG UGC UUA UCC AAC UCU G (dTdT)-3′. Mouse PD-L1 (NM 021893.3) was cloned into a pLVX-Puro lentivirus expression vector (Addgene, Watertown, MA, USA). Short hairpin RNAs (shRNAs) for mouse PD-L1 were prepared by cloning double-stranded oligonucleotides into a pLKO.1-Puro vector (Addgene). The target 21-mer sequences of mouse PD-L1 shRNAs were as follows: PD-L1 shRNA-1 5′-GTT TAC TAT CAC GGC TCC AAA-3′ and PD-L1 shRNA-2 5′-CAG GCG TTT ACT GCT GCA TAA-3′. The target sequence of scrambled shRNA was 5′-CAA CAA GAT GAA GAG CAC CAA-3′.

### PD-L1 overexpression and knockdown

Cells were transfected with PD-L1-expressing plasmid vector or PD-L1 siRNAs using jetPRIME transfection reagent (Polyplus, Illkirch-Graffenstaden, France) according to the manufacturer’s instructions. To generate mouse stable cell lines, pLKO.1-mCherry-mPD-L1 shRNA, pLKO.1-mCherry-scrambled shRNA, pLVX-mCherry-mPD-L1, and pLVX-mCherry were packaged into lentiviral particles by Lenti-X-293T packaging cells cotransfected with the viral packaging plasmids pVSG-G, BH10, and pcREV, and viral supernatants were harvested 32 to 40 h after transfection. LLC cells were infected with polybrene-added lentiviral supernatants and sorted on a FACSAria III cell sorter (BD Biosciences, Franklin Lakes, NJ, USA).

### RNA sequencing (RNA-seq)

A549 cells transfected with PD-L1-expressing vector and H460 cells transfected with PD-L1 siRNA were submitted to RNA-seq. RNA was extracted from the cells using TRIzol Reagent (Molecular Research Center, Cincinnati, OH, USA), and the quality and integrity of the total RNA were checked using NanoDrop 1000 3.7.1 spectrophotometer (Thermo Fisher Scientific, Waltham, MA, USA) and an Agilent 2100 Bioanalyzer (Agilent Technologies, Santa Clara, CA, USA). RNA-seq was performed by a NovaSeq 6000 system (Illumina, San Diego, CA, USA) at Theragen Bio Institute (Gyeonggi-do, Republic of Korea). To assess significant differences in gene expression under two different biological states, RNA-seq datasets were analyzed using Gene Set Enrichment Analysis (GSEA) software 4.2.3 and the Molecular Signatures Database (MSigDB) (Broad Institute, Cambridge, MA, USA).

### Quantitative real-time polymerase chain reaction (qRT-PCR)

Total mRNA was extracted from cells and tumor tissues and subjected to qRT-PCR analysis as described in the Supplementary Methods using primers with the sequences listed in Supplementary Table S1.

### Co-immunoprecipitation and Western blot

Co-immunoprecipitation and Western blotting were performed as described in the Supplementary Methods using the antibodies listed in Supplementary Table S2.

### Immunofluorescence staining

Cells were subjected to indirect immunofluorescence staining for EMT-related molecules as described in the Supplementary Methods.

### Enzyme-linked immunosorbent assay (ELISA)

TGFβ in culture supernatant was measured using an ELISA kit (R&D Systems) according to the manufacturer’s instructions.

### In vitro phosphatase assay

In vitro phosphatase assay was performed using recombinant human p-p38 protein (ab271647; Abcam, Cambridge, UK) as a substrate and recombinant human PPM1B (LS-G21072-20; LS Bio, Lynnwood, WA, USA) as a phosphatase in the presence or absence of recombinant human PD-L1 (156-B7; R&D Systems) as described in the Supplementary Methods.

### Pull-down assay

Pull-down assay was performed using GST-PD-L1 full length (FL), GST-PD-L1 extracellular domain (ECD), or GST-PD-L1 intracellular domain (ICD) His-tagged PPM1B as described in Supplementary Methods.

### Luciferase reporter assay

A 129 bp region of the human TGFβ promoter that includes ATF2 (TGA-GTCA) and c-Jun-binding motif (TGA-GTCA) was cloned into the luciferase-expressing plasmid pGL4.24-luc2P (E842A; Promega, Madison, WI, USA). After transfections of cells with PD-L1 siRNA or PPM1B siRNA and then with the cloned luciferase-expressing plasmid, luciferase reporter assay was performed as described in the Supplementary Methods.

### Cell proliferation, migration, and invasion assay

Cell proliferation, migration, and invasion was analyzed by a Cell Count Kit-8 (CCK-8) assay and cell migration and invasion were evaluated using a wound-healing assay and Transwell migration and invasion assay as described in the Supplementary Methods.

### Mice and in vivo experiments

Six- to seven-week-old specific-pathogen-free female Balb/c-nu mice were purchased from Orient Bio (Gyeonggi-do, Republic of Korea) and maintained under specific-pathogen-free conditions in accordance with the animal care guidelines approved by the Institutional Animal Care and Use Committee of Seoul National University (Approval No. SNU-220303−5) and Seoul National University Hospital (SNUH) (Approval No. 22−0082-S1A0(1)).

Stable PD-L1-overexpressing- or PD-L1-knockdown LLC cells and their respective control cells were subcutaneously implanted (2 × 10^5^ cells/mouse, respectively) into the mammary fat pad of female Balb/c-nu mice. The primary tumor volume was measured once every 2 to 3 days using calipers and calculated using the following the formula: tumor volume (mm^3^) = (longest diameter × shortest diameter^2^)/2. In the other model, 5 × 10^5^ cells in 100 μL sterile phosphate-buffered saline (PBS) were injected into the tail vein. At the endpoint, the lungs were excised promptly after euthanasia, and the number of metastatic nodules on the lobes was counted. The right lung lobes were fixed with 4% paraformaldehyde solution and embedded in paraffin for histological analyses. The left lobes were homogenized to analyze protein and RNA expression.

### ICI cohorts and analysis of patient tumor tissue

Two cohorts of patients with NSCLC receiving ICI therapy (PD-1 or PD-L1 blockade) were included in this study. In the RNA-seq cohort (*n* = 234), tumor tissues were obtained prior to ICI therapy at Samsung Medical Center (SMC) and SNUH and subjected to RNA-seq. RNA was extracted from fresh or formalin-fixed, paraffin-embedded (FFPE) tumor tissues using an AllPrep DNA/RNA Mini Kit (Qiagen, Hilden, Germany). The library was constructed using an RNA Access Library Prep Kit (Illumina). RNA-seq reads were aligned using STAR 2.5.2b, and gene expression levels were quantified using RSEM v1.3.0 with batch effect correction.

In the immunohistochemistry (IHC) cohort (*n* = 90), resected tumor tissues from patients, who had undergone surgical resection at SNUH before ICI therapy were reviewed and representative FFPE tumor blocks were subjected to IHC as described in the Supplementary Methods.

The characteristics of patients from each cohort are summarized in Supplementary Tables S3 and S4. The treatment response was evaluated based on the Response Evaluation Criteria in Solid Tumors version 1.1 and was classified as complete response (CR), partial response (PR), stable disease, or progressive disease (PD). The study was conducted in accordance with guidelines approved by institutional review boards of SMC (SMC-2013−10−112 and SMC-2018−03−130) and SNUH (H-1905−115−1035), and all participants provided written informed consent.

### Assessment of EMT score and deconvolution of tumor-infiltrating immune cells

To compute the EMT score, we used cancer-specific EMT signatures consisting of 315 genes for tumor tissue (including 145 epithelial and 170 mesenchymal genes) and 218 genes for the cell line (including 170 epithelial and 48 mesenchymal genes) (listed in Supplementary Table S5) [36]. The empirical cumulative distribution function was estimated for the epithelial and mesenchymal gene sets. The two-sample Kolmogorov-Smirnov test was employed to compute the difference between the mesenchymal and epithelial empirical cumulative distribution functions. Then the two-sample Kolmogorov-Smirnov score was taken as the EMT score. A positive EMT score reflects a more mesenchymal phenotype, whereas a negative EMT score reflects a more epithelial phenotype.

To assess the tumor-infiltrating immune cell composition in the ICI RNA-seq cohort, the relative abundances of 22 immune cell types were analyzed using a gene signature matrix (LM22) according to the Cell-Type Identification by Estimating Relative Subsets of RNA Transcripts algorithm (http://cibersort.stanford.edu/). Gene signatures for the cytotoxic T lymphocyte (CTL) response, regulatory T-cells (Tregs), M1 macrophages, and M2 macrophages were based on the published gene sets listed in Supplementary Table S5. Signature scores were defined as the mean of Z-score of log_2_(fold-change) among all genes in each signature.

### Public database analysis

The Level 3 RNA-seq data of lung adenocarcinoma (LUAD) and lung squamous cell carcinoma (LUSC) from The Cancer Genome Atlas (TCGA) projects were downloaded from TCGA data portal (http://portal.gdc.cancer.gov/) using the “TCGABiolinks” R package. TCGA datasets were analyzed using GSEA software 4.2.3 and MSigDB (Broad Institute). The EMT score was computed as described above.

### Statistical analysis

Data are presented as the mean ± standard error of the mean and were analyzed using GraphPad Prism 9 statistical software (GraphPad Software, San Diego, CA, USA). Significant differences between two groups among multiple groups were analyzed by the unpaired two-tailed Student’s *t*-test and one-way analysis of variance followed by Dunnett’s multiple comparison test, respectively. Statistical significance and correlation coefficients in the correlation analysis were calculated based on Spearman’s correlation analysis. A *p* value of < 0.05 was considered statistically significant.

## Results

### PD-L1 intrinsically promotes EMT in lung cancer cells

**Fig. 1:**
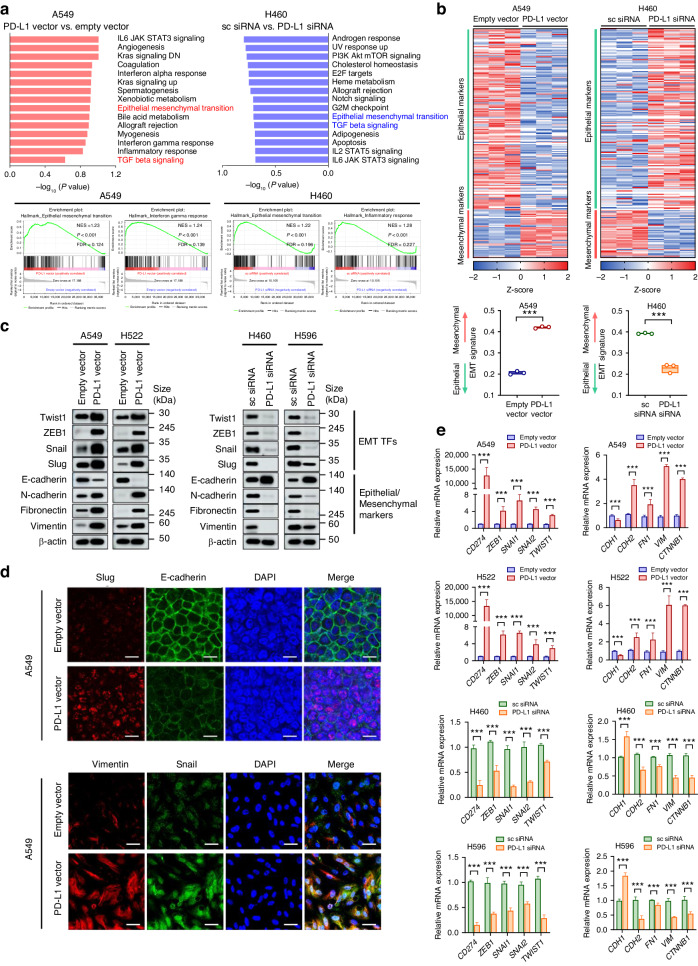
Expression of PD-L1 intrinsically promotes EMT in lung cancer cells. The human NSCLC cell lines A549 and H522 were transfected with PD-L1-expressing or empty vectors, and H460 and H596 were transfected with PD-L1 siRNA or scrambled siRNA. At 24 h after plasmid vector transfection and 48 h after siRNA transfection, cells were subjected to RNA and protein extraction for further analysis. **a** The figure depicts hallmark gene sets enriched in PD-L1-overexpressing A549 compared to control as well as those downregulated in PD-L1-knockdown H460 compared to control. **b** Heatmap showing differences in mRNA expression of 170 epithelial markers and 48 mesenchymal markers in A549 cells with PD-L1 overexpression versus control and H460 cells with PD-L1 knockdown versus control. **c**, **d** Protein expression of EMT markers was analyzed by Western blotting and immunofluorescence staining. Scale bar = 10 μm (upper), 20 μm (lower). **e** mRNA expression of EMT markers was analyzed by qRT-PCR. Histograms represent values normalized to control. Data are presented as mean ± S.E.M. of three independent experiments. *p < 0.05, **p < 0.01, and ***p < 0.001.

To investigate the intrinsic PD-L1 function in lung cancer cells, basal expression levels of PD-L1 were compared among human NSCLC cell lines (Supplementary Fig. S1A). Then, A549 and H522 cells with low basal PD-L1 expression were transfected with PD-L1-expressing or control vectors, and H460 and H596 cells with high basal PD-L1 expression were transfected with PD-L1 siRNA or scrambled siRNA. A549 and H460 cells were subjected to RNA-seq for whole-transcriptomic analysis. GSEA revealed that several pathways were differently enriched according to the PD-L1 expression level. The EMT pathway was upregulated by PD-L1 overexpression in A549 cells and downregulated by PD-L1 knockdown in H460 cells (Fig. 1a). A heat map for EMT signature genes showed decreased epithelial genes and increased mesenchymal genes in A549 with PD-L1 overexpression, and increased epithelial genes and decreased mesenchymal genes in H460 with PD-L1 knockdown (Fig. 1b). Consistently, the protein and mRNA levels of EMT-related transcription factors and mesenchymal markers including Twist1, ZEB1, Snail, Slug, N-cadherin, Fibronectin, and Vimentin were increased and those of the epithelial marker E-cadherin were decreased in NSCLC cells with higher PD-L1 expression (Fig. 1c−e and Supplementary Fig. S1B). In TCGA analysis, PD-L1-high LUAD and LUSC exhibited a significantly enriched EMT pathway compared to PD-L1-low LUAD and LUSC (Supplementary Fig. S2). These data suggest that PD-L1 expression by tumor cells intrinsically promotes the EMT phenotype in NSCLC.

### Tumor cell-intrinsic PD-L1 promotes EMT via TGFβ production

**Fig. 2:**
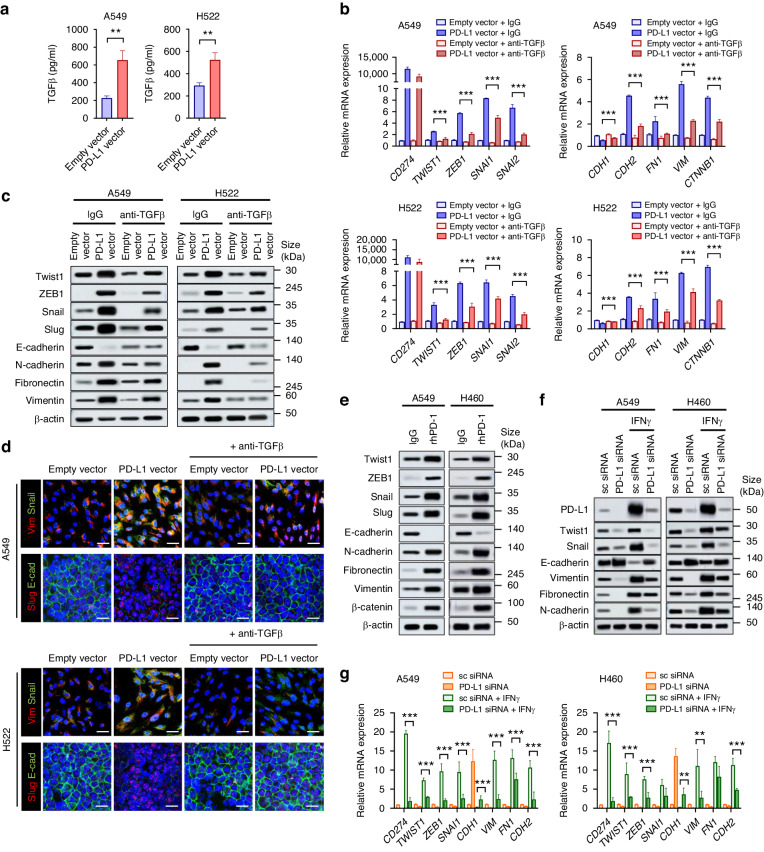
PD-L1 promotes EMT via TGFβ production. **a** A549 and H522 cells were transfected with PD-L1-expressing or empty vector. At 24 h after transfection, TGFβ production in the culture supernatant was measured using an ELISA. **b**–**d** A549 and H522 cells were transfected with PD-L1-expressing or empty vector in the presence or absence of anti-TGFβ neutralizing antibody (1 μg/mL). At 24 h after transfection, mRNA and protein expression for EMT markers were analyzed by qRT-PCR (**b**), Western blotting (**c**), and immunofluorescence staining (**d**). Scale bar = 20 μm. **e** A549 and H522 cells were incubated with recombinant human PD-1 (1 μg/mL) for 24 h, and the expression of EMT markers was analyzed by Western blotting. **f**, **g** A549 and H460 cells were treated with IFNγ (100 ng/mL) with or without PD-L1 knockdown, and 48 h later, protein and mRNA expression of EMT markers was analyzed by Western blotting and qRT-PCR. Histograms represent values normalized to control. Data are presented as mean ± S.E.M. of three independent experiments. *p < 0.05, **p < 0.01, and ***p < 0.001.

We hypothesized that TGFβ might be involved in tumor cell-intrinsic PD-L1-induced EMT because PD-L1-knockdown H460 displayed a significant decrease in TGFβ signaling (Fig. 1a). PD-L1-overexpressing A549 and H522 cells showed higher secretion of TGFβ (Fig. 2a), and anti-TGFβ neutralizing antibody treatment partially restored the PD-L1-induced EMT phenotype in A549 and H522 cells (Fig. 2b–d and Supplementary Fig. S3A). These data indicate that tumor cell-intrinsic PD-L1 may induce EMT by TGFβ production.

To determine whether tumor cell-intrinsic PD-L1 function could be stimulated by PD-1/PD-L1 interaction, A549 and H460 cells were treated with rhPD-1, which enhanced EMT in phenotype (Fig. 2e). Cancer cells are often exposed to IFNγ within the TME, and IFNγ is a well-known strong inducer of PD-L1 expression [1]. IFNγ induced PD-L1 expression and increased EMT-related molecule expression, and the latter was partially restored by PD-L1 knockdown (Fig. 2f, g and Supplementary Fig. S3B, C). These results suggest that PD-L1 is involved in IFNγ-induced EMT and that IFNγ-induced PD-L1 within the TME might contribute to EMT of lung cancer cells.

### PD-L1 directly interacts with and inhibits the protein phosphatase PPM1B and thereby activated p38 MAPK and TGFβ production

**Fig. 3:**
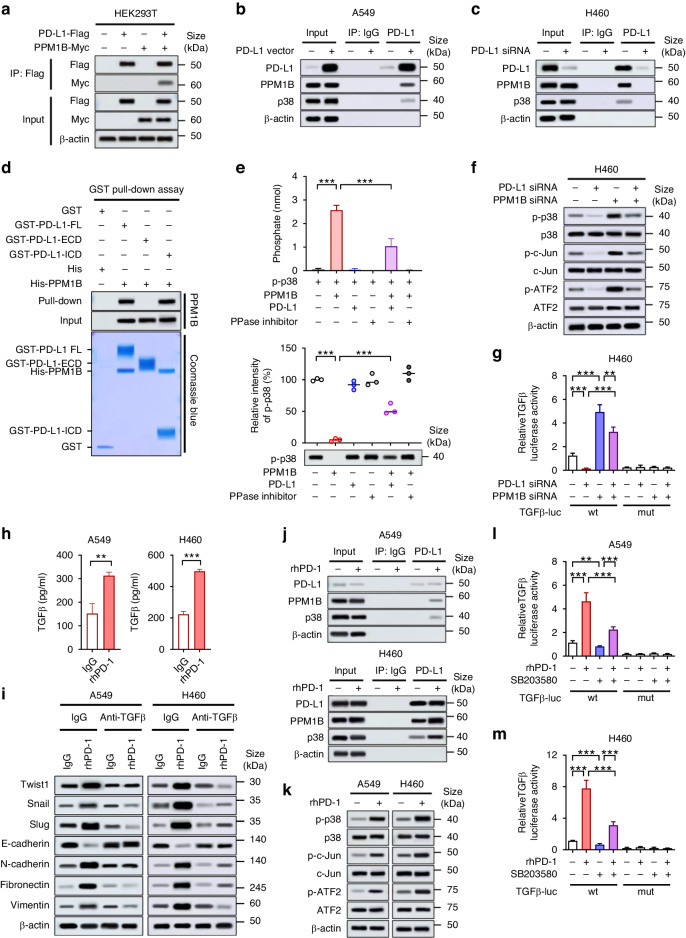
PD-L1 directly interacts with PPM1B and inhibits its phosphatase activity, thereby activating p38 MAPK pathway and TGFβ production. **a** HEK293T cells were transfected with Flag-tagged PD-L1 and Myc-tagged PPM1B for 48 h and then subjected to immunoprecipitation followed by immunoblotting. **b**, **c** A549 and H460 cells were transfected with PD-L1 overexpressing vector or PD-L1 siRNA. At 24 h of transfection, cells were submitted to co-immunoprecipitation assay with PD-L1 to evaluate the protein complex formation with PPM1B and p38 MAPK. **d** Full length (FL), extracellular domain (ECD), and intracellular domain (ICD) of GST-tagged PD-L1 were purified from *E. coli*. GST pull-down assays were performed using 1 μg of each indicated protein. **e** In vitro phosphatase assay was performed using recombinant p-p38 as a substrate and recombinant PPM1B as a phosphatase in the presence and absence of recombinant PD-L1 and phosphatase inhibitor (PPase inhibitor). Phosphatase activity was evaluated by measurement of free phosphate (upper) and immunoblotting for p-p38 (lower). Relative intensity of p-p38 was estimated on immunoblot. **f** H460 cells were transfected with PD-L1 siRNA and/or PPM1B siRNA. At 24 h after transfection, cell were submitted to Western blotting for indicated molecules. **g** H460 cells were transfected with PD-L1 siRNA and/or PPM1B siRNA for 24 h and then transfected with luciferase-expressing vector with wild-type (wt) promoter sequence of TGFβ containing ATF2- and c-Jun-binding motif. At 12 h after transfection, luciferase activity was measured. As a negative control, the plasmid with truncated mutation (mut) of the TGFβ promoter sequence were used. **h** A549 and H460 cells were incubated with rhPD-1 (1 μg/ml) for 24 h, and TGFβ production in the culture supernatant was measured using an ELISA. **i** A549 and H460 cells were treated with rhPD-1 (1 μg/ml) in the presence or absence of anti-TGFβ neutralizing antibody (1 μg/mL). At 24 h after treatment, total protein was extracted and submitted to Western blotting for EMT markers. **j**, **k** After 24 h treatment of rhPD-1, protein complex of endogenous PD-L1, PPM1B, and p38-MAPK was analyzed by co-immunoprecipitation assay (**j**), and phosphorylation of p38, c-Jun, and ATF2 was evaluated using Western blotting (**k**) in A549 and H460 cells. **l**, **m** A549 and H460 cells were treated with rhPD-1 in the presence or absence of p38 inhibitor (SB203580, 10 μM). Cells were then transfected with TGFβ-luc vector and at the 12 h of transfection luciferase activity of promoter sequence of TGFβ containing ATF2- and c-Jun-binding motif were measured. Data are presented as mean ± S.E.M. of three independent experiments. *p < 0.05, **p < 0.01, and ***p < 0.001.

To identify how PD-L1 induces TGFβ production, we investigated candidate proteins interacting with PD-L1. According to Gao Y et al. [17], proteins that potentially associate with PD-L1 were identified, and one of them was the protein phosphatase Mg2 + /Mn2+ dependent 1B (PPM1B) [37, 38]. In co-immunoprecipitation assay, PPM1B and PD-L1 were associated with each other (Fig. 3a). In addition, endogenous PPM1B was found to form protein complexes with overexpressed PD-L1 in A549 cells and endogenous PD-L1 in H460 cells, along with endogenous p38 MAPK (Fig. 3b, c). Pull-down assay showed the direct interaction of PPM1B and cytoplasmic domain of PD-L1 (Fig. 3d). The direct interaction of PD-L1 with PPM1B raised a possibility that PPM1B might play a role in the PD-L1-dependent regulation of p38 MAPK. Indeed, in in vitro phosphatase assay using recombinant p-p38 as substrate, PD-L1 inhibited PPM1B activity (Fig. 3e). These findings suggested that PD-L1, by binding to PPM1B, can inhibit PPM1B-mediated dephosphorylation of p38 MAPK and thus preserve the p38 MAPK activity. In PD-L1-high H460 and H596 cells, PD-L1 knockdown led to decreased p38 phosphorylation, which was recovered by concomitant PPM1B knockdown (Fig. 3f and Supplementary Fig. S3D). In addition, the phosphorylation of ATF2 and c-Jun, which are downstream molecules of p38 MAPK and transcription factors of TGFβ [39, 40], were decreased by PD-L1 knockdown and recovered by concomitant PPM1B knockdown along with phosphorylation of p38 (Fig. 3f and Supplementary Fig. S3D). Consistently, luciferase reporter assay showed that transcription of TGFβ was suppressed by PD-L1 knockdown in H460 and H596 cells, which was restored by concomitant PPM1B knockdown (Fig. 3g and Supplementary Fig. S3E). Together, these findings suggest that PD-L1 may induce transcription of TGFβ by inhibiting PPM1B and subsequently sustaining p38 MAPK activity and ATF2 and c-Jun activities.

Meanwhile, rhPD-1 treatment also increased TGFβ production in NSCLC cells (Fig. 3h), and anti-TGFβ neutralizing antibody restored the EMT phenotype promoted by rhPD-1 treatment (Fig. 3i and Supplementary Fig. S3F). rhPD-1 treatment increased interactions of PD-L1 with PPM1B and p38, as well as the phosphorylation of p38, c-Jun, and ATF2 in A549 and H460 cells (Fig. 3j, k). Transcriptional activity of TGFβ presumably caused by c-Jun and ATF2 was increased upon rhPD-1 treatment, which was restored by p38 inhibitor (SB203580) (Fig. 3l, m). These findings suggest that p38 MAPK pathway may be involved in TGFβ production by PD-1/PD-L1 interaction in NSCLC cells.

### PD-L1 promotes proliferation, migration, and invasion of lung cancer cells

**Fig. 4:**
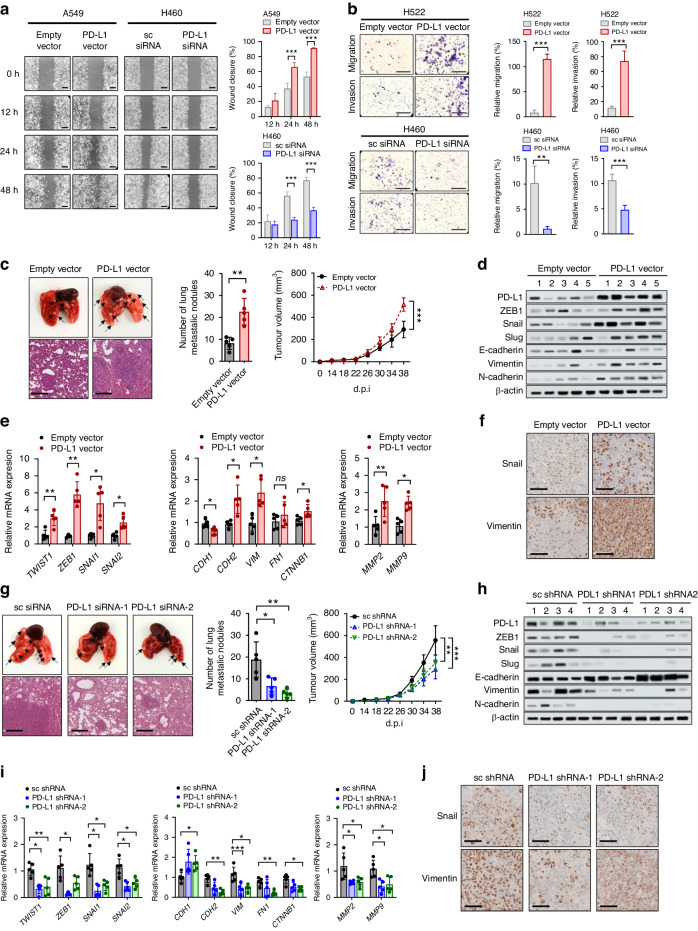
PD-L1 overexpression enhances cell migration and invasion in vitro and promotes tumor progression and metastasis in vivo independently of anti-tumor immunity. A549 and H522 cells were transfected with PD-L1-expressing or empty vector, and H460 and H596 cells were transfected with PD-L1 siRNA or scrambled siRNA. Then the cells were submitted to (**a**) a wound healing assay, and (**b**) cell migration invasion assay using Transwells. The wound closure rate was calculated using ImageJ. Scale bar = 1000 μm. The traversed cells after 24 h incubation were counted using ImageJ. Scale bar = 1000 μm. Histograms represent values normalized to control. PD-L1-overexpressing or vector control LLC cells and PD-L1-knockdown or shRNA control LLC cells were injected into the mammary fat pads of Balb/c-nu mice. **c**, **g** The primary tumor volume was measured with calipers every 2 or 3 days and calculated using the standard formula. Lung metastases were dissected 38 days after cancer cell injection, and tumor lung nodules (arrows) were counted. Representative hematoxylin- and eosin-stained images of lung tissues showed metastatic lesions. **d**, **e**, **h**, **i** mRNA and protein expression of EMT markers in lung tissues were analyzed by qRT-PCR and Western blotting. Numbers in (**d**) and (**h**) denotes individual mouse. **f**, **j** Representative IHC staining images for Snail and vimentin of lung tumors. Data in histograms are presented as mean ± S.E.M. of three independent experiments. *p < 0.05, **p < 0.01, and ***p < 0.001.

In vitro assays were performed to determine whether the intrinsic function of PD-L1 affects tumor aggressiveness. Cell proliferation was significantly increased in A549 and H522 cells with PD-L1 overexpression but decreased in H460 and H596 cells with PD-L1 knockdown (Supplementary Fig. S4A, B). In addition, PD-L1 overexpression promoted cell migration and invasion of A549 and H522 cells, whereas PD-L1 knockdown inhibited cell migration and invasion of H460 and H596 cells (Fig. 4a, b and Supplementary Fig. S4C, D).

### PD-L1 promotes tumor metastasis in vivo independently of anti-tumor immunity

The results of the in vitro studies suggested that the tumor cell-intrinsic function of PD-L1 contributes to the aggressiveness of lung cancer independently of anti-tumor immunity. For in vivo examination of this phenomenon, mouse lung cancer LLC cells with stably overexpressed or knock-down PD-L1 were transplanted into immunodeficient BALB/c-nu mice via mammary fat pad and tail vein injection. PD-L1 overexpression promoted the EMT phenotype of LLC cells in vitro (Supplementary Fig. S5). In the mammary fat pad injection model, tumor growth in situ and lung metastatic nodules were significantly increased in mice bearing LLC cells with PD-L1 overexpression (Fig. 4c), and decreased in mice bearing LLC cells with PD-L1 knockdown (Fig. 4g). In addition, expression of EMT markers, matrix metalloproteinases 2 and 9 was higher in lung tissues (harboring metastatic tumors) from mice bearing LLC cells with PD-L1 overexpression and lower in lung tissues from mice bearing LLC cells with PD-L1 knockdown (Fig. 4d–f and Fig. 4h–j). Consistent findings were observed in the tail vein injection model (Supplementary Fig. S6). Together, these results suggest that the tumor cell-intrinsic function of PD-L1 may increase tumor progression and metastasis independently of anti-tumor immunity or its extrinsic function as an immune checkpoint.

### EMT is associated with poor response to ICI therapy and correlates with immunosuppressive microenvironment in PD-L1-high NSCLC

**Fig. 5:**
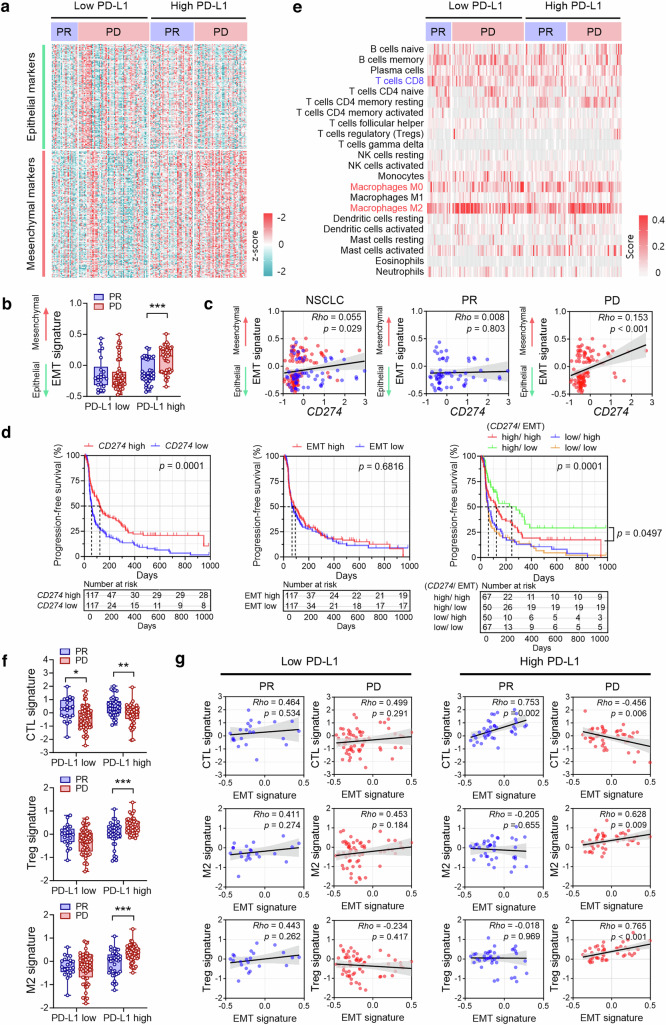
EMT predicts a poor response to ICI therapy in PD-L1-high NSCLC (RNA-seq cohort). Tumor tissues from 234 patients with NSCLC prior to ICI therapy were subjected to RNA-seq. **a** Heatmap shows the mRNA expression of 145 epithelial markers and 170 mesenchymal markers in patients with NSCLC treated with ICIs. The patients were divided into PD-L1-high and PD-L1-low groups based on the median PD-L1 expression and response to ICI therapy (PR, partial response; PD, progressive disease). **b** Differences in EMT scores between patients showing PR and PD to ICI therapy in the PD-L1-low and PD-L1-high groups, respectively. **c** Correlation between PD-L1 (*CD274*) expression and EMT score in patients showing PR (blue dot) and PD (red dot). **d** All patients with ICI therapy (including those showing PR, stable disease, and PD) were divided into low versus high groups in terms of PD-L1 expression and the EMT score according to the median values. Kaplan–Meier analysis of PFS after ICI therapy was performed according to PD-L1 expression (left), EMT score (middle), and the combined status of PD-L1 expression and the EMT score (right). The survival difference was analyzed using the log-rank test. **e** To assess the immune cell composition among the groups, 22 immune cell types were analyzed using the CIBERSORTx algorithm. **f** Differences in CTL, Treg, and M2 macrophage signatures between patients showing PR and PD to ICI therapy in the PD-L1-low and PD-L1-high groups, respectively. **g** Correlations between CTL, M2 macrophage, and Treg signatures and EMT scores in patients with PR and PD to ICI therapy in the PD-L1-low and PD-L1-high groups, respectively. Data in the histogram are presented as mean ± S.E.M. Correlations among variables were calculated using Spearman’s correlation test. *p < 0.05, **p < 0.01, and ***p < 0.001.

To investigate the role of PD-L1-induced EMT in cancer immunotherapy, two cohorts of patients with NSCLC (RNA-seq and IHC cohorts) treated with ICIs against the PD-1/PD-L1 pathway were utilized. In the RNA-seq cohort, the EMT signature was higher in patients showing PD than in those showing a PR among patients with PD-L1-high NSCLC, but there were no differences in the EMT signature according to the therapeutic response to ICIs among patients with PD-L1-low NSCLC (Fig. 5a, b). Moreover, the EMT score and individual EMT-related molecule expression were significantly positively correlated with PD-L1 expression in patients with PD but not in those with a PR (Fig. 5c and Supplementary Fig. S7, S8). In the Kaplan−Meier analysis, high PD-L1 (*CD274*) expression (defined as above median) was significantly associated with prolonged progression-free survival (PFS), but the EMT score was not (Fig. 5d). However, a high EMT score (defined as above median) was associated with shorter PFS after ICI therapy in patients with PD-L1-high NSCLC, but not in patients with PD-L1-low NSCLC (Fig. 5d). High expression of individual EMT-related molecules was also related to shorter PFS after ICI therapy in patients with PD-L1-high NSCLC (Supplementary Fig. S9).

To investigate the effect of EMT on the tumor immune microenvironment in NSCLC, the immune cell composition in NSCLC tissues was analyzed using the CIBERSORTx algorithm and immune cell signature. Among patients with PD-L1-high NSCLC, patients with PD after ICI therapy displayed decreased CD8 T-cell and CTL signature, increased M2 macrophages and M2 signature, and increased Tregs and Treg signature compared to patients who achieved a PR (Fig. 5e, f and Supplementary Fig. S10). These relationships were not observed in patients with PD-L1-low NSCLC except for *CD8A* expression and the CTL signature. Notably, correlation analysis revealed a significant negative correlation between CTL signature and EMT score and significant positive correlations between M2 macrophages or Tregs and EMT scores in patients who had PD-L1-high NSCLC with PD (Fig. 5g). These correlations were not observed in patients with PD-L1-low NSCLC irrespective of the response to ICI therapy (Fig. 5g).

Taken together, these findings suggest that EMT in PD-L1-high NSCLC may contribute to poor response and unfavorable clinical outcomes after ICI therapy probably by affecting the TME, promoting its shift to immunosuppression.

### EMT-related molecule expression in tumor cells predicts poor response to ICI therapy and unfavorable clinical outcomes in patients with PD-L1-high NSCLC

**Fig. 6:**
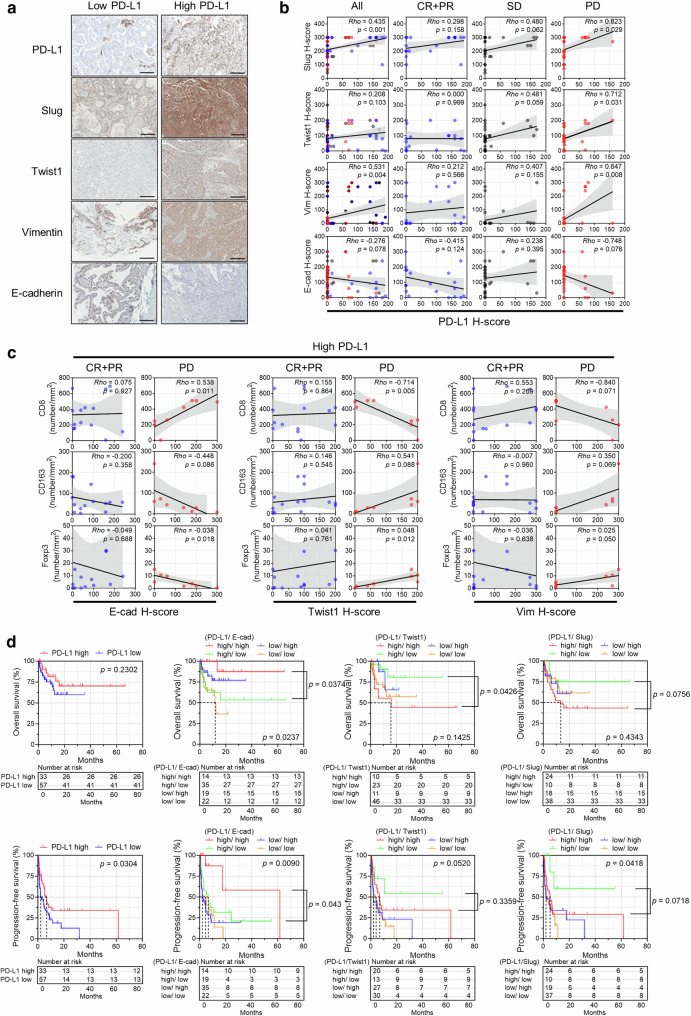
EMT in tumor cells is a predictive biomarker for a poor clinical outcome after ICI therapy in patients with PD-L1-high NSCLC (IHC cohort). Tumor tissues from 90 patients with NSCLC who underwent ICI therapy were subjected to IHC. The patients were divided into PD-L1-low and PD-L1-high groups using a cutoff of a PD-L1 TPS of 1 as well as EMT molecule-low and EMT molecule-high groups using a cutoff of the H-score determined from the Euclidean distance on the receiver operating characteristic curve. **a** Representative IHC staining images of Slug, Twist1, vimentin, and E-cadherin in patients with PD-L1-low and PD-L1-high NSCLC. **b** Correlations between the H-scores of PD-L1 and EMT molecules in all patients, patients showing a complete response (CR) and partial response (PR), patients showing stable disease (SD), and patients showing progressive disease (PD). **c** Correlations between the H-score of EMT molecules and the numbers of CD8^+^, CD163^+^, and FOXP3^+^ cells in patients showing CR plus PR and PD, respectively, in PD-L1-high NSCLCs. **d** Kaplan−Meier analysis of overall survival and PFS after ICI therapy according to PD-L1 expression and the combined status of PD-L1 expression and EMT molecule expression. The survival difference was analyzed using the log-rank test. Correlations among variables were calculated using Spearman’s correlation test.

Analysis of the IHC cohort showed positive correlations between Slug, Twist1, or vimentin expression and PD-L1 expression and a negative correlation between E-cadherin expression and PD-L1 expression by tumor cells in patients with PD after ICI therapy (Fig. 6a, b). In PD-L1-high (defined as a TPS ≥ 1) NSCLC, the mesenchymal phenotype (i.e., increased Twist1 or vimentin expression and decreased E-cadherin expression) was associated with a lower number of CD8^+^ tumor-infiltrating lymphocytes (TILs) and a higher number of FOXP3^+^ TILs and CD163^+^ cells in patients showing PD (Fig. 6c). This relationship was not observed in patients with PD-L1-low NSCLC. Although EMT itself was not associated with the prognosis after ICI therapy (Supplementary Fig. S12), high expression of Twist1 and Slug, and low expression of E-cadherin were associated with shorter overall survival and PFS in patients with PD-L1-high NSCLC (Fig. 6d). These findings validated the results in the RNA-seq cohort and suggest that the protein expression of EMT-related markers by tumor cells could be a predictive biomarker for ICI therapy in patients with PD-L1-high NSCLC.

## Discussion

We demonstrated that cell-intrinsic PD-L1 signaling induces EMT in NSCLC via TGFβ, thereby promoting tumor progression and metastasis in vitro and in vivo. This is the first study to explore the relationship between EMT and the ICI response in the context of PD-L1 expression and has demonstrated that EMT may play a role as a predictive biomarker for ICI therapy in patients with PD-L1-high NSCLC.

EMT is a functional consequence of tumor cell-intrinsic PD-L1 signaling [9], but its mechanism remains to be elucidated. Cell-intrinsic PD-L1 signaling activated PI3K/Akt pathways in nasopharyngeal carcinoma cells, thereby promoting EMT [28]. In a study of breast cancer, PD-L1 inhibited GSK3β function by binding and inhibiting the tyrosine phosphatase PTP1B, thus preventing the GSK3β-mediated degradation of Snail [12]. In a study of NSCLC, PD-L1 induced EMT by activating the WASP-interacting protein and β-catenin pathways [14]. This study demonstrated that PD-L1 overexpression promoted or enhanced EMT by TGFβ upregulation. Because TGFβ signaling pathway is regulated by PD-L1 expression (Fig. 1a) and the TGFβ-Smad signaling pathway induces variable EMT transcription factors including the Snail family (Snail, Slug), ZEB family (ZEB1, ZEB2), and basic helix-loop-helix family (Twist and others) [41], we hypothesized that TGFβ might be involved in PD-L1-induced EMT of NSCLC. In a previous study using murine melanoma model, it was reported that tumor cell-intrinsic PD-L1 and TGFβ upregulated each other in a bidirectional way and was involved in EMT [29]. However, the mechanism by which cell-intrinsic PD-L1 upregulated TGFβ remains unknown. In this study, we demonstrate that PD-L1 binds to and suppresses the phosphatase PPM1B and thereby contributes to sustained activation of p38 MAPK and upregulation of TGFβ. This mechanistic insight could underline the potential utility of bispecific antibodies targeting TGFβ and PD-L1 under clinical trials in solid tumors, including NSCLC [42]. Meanwhile, TGFβ has been studied as a biomarker and target for ICI therapy. TGFβ signaling in fibroblasts of the TME attenuated the response to ICI therapy by excluding T-cell infiltration into the tumor [43]. The present study showed that TGFβ signaling in cancer cells induced by intrinsic PD-L1 function can contribute to EMT and immune evasion of cancer, compromising the responsiveness to ICI therapy (Supplementary Fig. S12).

Bioinformatics-based pan-cancer multi-omics analysis revealed a relationship between EMT and immune evasion and the potential utility of combined EMT and immune cytolytic activity as a predictor of ICI therapy in several cancers [34]. However, limited data from a small cohort are available regarding the significance of EMT in the responsiveness to ICI in patients with NSCLC [35]. Given that tumoral PD-L1 expression is the most widely used practical biomarker for ICI therapy in NSCLC, and because PD-L1 and EMT exhibit crosstalk between each other as shown by previous studies and the present study, the potential role of EMT as a predictive biomarker for ICI therapy needs to be explored in the context of PD-L1 expression. Using two independent cohorts of patients with NSCLC undergoing ICI therapy, we demonstrated that the significance of EMT as a predictive biomarker was dependent on the PD-L1 expression status. The EMT signature was significantly elevated in patients with PD compared to who showed a response to ICI therapy in PD-L1-high NSCLC but not in PD-L1-low NSCLC. Survival after ICI therapy was worse in patients with EMT-high/PD-L1-high NSCLC than in patients EMT-low/PD-L1-high NSCLC, but there were no differences in survival according to EMT in patients with PD-L1-low NSCLC. These findings indicate that EMT may serve as a predictive (unfavorable) biomarker for ICI therapy in PD-L1-high but not PD-L1-low NSCLC. These findings also suggest that EMT may be a potential therapeutic target to improve ICI efficacy in PD-L1-high NSCLC.

Previous studies have shown that EMT signatures are associated with a unique TME of NSCLC characterized by elevated inflammatory signals, IFNγ, multiple coinhibitory and costimulatory molecule expression, and higher infiltration of CD8^+^ and CD4^+^ T-cells, Tregs, and macrophages independently of the tumor mutational burden [24, 25, 34]. Sarcomatoid carcinoma of the lung showed the highest PD-L1 expression among all NSCLC types and was infiltrated by a higher number of T-cells compared to non-sarcomatoid NSCLC [22]. These findings suggest that EMT might contribute to immunosuppression and immune evasion in NSCLC within an inflamed TME. Several mechanisms underlying EMT-induced immunosuppression have been reported in melanoma, breast cancer, and NSCLC [30, 32, 33, 44–47]. Soluble and insoluble factors including TGFβ, thrombospondin 1, secreted phosphoprotein 1, colony-stimulating factor 1, and CD73 are expressed by melanoma and breast cancers with EMT and promote Treg infiltration, inhibition of dendritic cell function, and polarization of tumor-associated macrophages (TAMs) into M2 macrophages [30, 33]. NSCLC with a mesenchymal phenotype shows immunoproteasome deficiency and subsequent defects in antigen presentation [44]. ZEB1 induces CD47 expression on invading NSCLC cells to drive M2 polarization of adjacent TAMs [45]. In our ICI cohorts, EMT was consistently correlated with a decreased CTL signature or CD8^+^ T-cells and increased M2 macrophages and Tregs in patients with PD after ICI therapy but not in those with a response to ICI. Notably, these relationships were observed in PD-L1-high NSCLC but not in PD-L1-low NSCLC. These findings suggest that EMT may contribute to the immunosuppression in PD-L1-high NSCLC rather than PD-L1-low NSCLC and thus predict a poor response to ICI therapy in PD-L1-high NSCLC. These observations also emphasize the crosstalk between PD-L1, EMT, and immunosuppression in NSCLC and the predictive role of EMT for ICI therapy in the context of PD-L1 expression (Supplementary Fig. S12).

The present study using human and mouse NSCLC cells clearly showed that PD-L1 induced the EMT phenotype as well as tumor growth, invasion, and metastasis in vitro and in vivo, emphasizing the tumor-promoting role of PD-L1 independent of the immune system. Tumoral PD-L1 expression can be endogenously induced by oncogenic signaling or PD-L1 gene alterations in tumor cells or adaptively by IFNγ secreted from tumor-infiltrating immune cells in the TME [1]. In the present study, PD-L1 expression by a plasmid vector (mimicking endogenous PD-L1 expression), as well as PD-L1 expression by IFNγ treatment (mimicking adaptive PD-L1 expression), affected the EMT phenotype. A few studies have shown that IFNγ induces EMT in prostate, renal, and lung cancer [48–50]. The current study is the first to show that IFNγ might contribute to EMT of NSCLC by PD-L1 induction and its intrinsic function.

Tumor cell-intrinsic PD-L1 signaling can occur with or without PD-1 ligation, and PD-L1 localization (i.e., surface, cytoplasm, and/or nucleus) can affect cell-intrinsic PD-L1 signaling [9, 17]. In this study, PD-L1-induced EMT was observed by PD-L1 overexpression itself and recombinant PD-1 treatment, which suggests that both PD-1-independent and PD-1-dependent pathways might be involved in PD-L1-induced EMT. In TME, ligation of PD-L1 on tumor cells by PD-1 on immune cells, particularly on Tregs (which highly express PD-1), and activated or exhausted T-cells, might be involved in the EMT of tumor cells. In addition, TGFβ secreted by Tregs or M2 macrophages, which are rich in patient with PD-L1-high/EMT-high NSCLC with PD after ICI therapy, could further enhance the EMT of tumor cells, requiring further studies (Supplementary Fig. S12).

In summary, this study demonstrates that tumor cell-intrinsic PD-L1 function induces EMT by TGFβ and promotes progression and metastasis of NSCLC in vitro and in vivo and that the predictive role of EMT in ICI therapy in patients with NSCLC depends on the PD-L1 expression status. EMT predicts an unfavorable response and outcome after ICI therapy in patients with PD-L1-high NSCLC and could be a therapeutic target to improve the efficacy of immunotherapy.

### Supplementary information

Supplementary Figures

Supplementary Methods and Tables

## Data Availability

The data generated in this study are available in the article and its supplementary data files or upon request from the corresponding author.
