# Correction: Epithelial−mesenchymal transition induced by tumor cell-intrinsic PD-L1 signaling predicts a poor response to immune checkpoint inhibitors in PD-L1-high lung cancer

**DOI:** 10.1038/s41416-024-02798-1

**Published:** 2024-07-30

**Authors:** Hyein Jeong, Jaemoon Koh, Sehui Kim, Seung Geun Song, Soo Hyun Lee, Youngjoo Jeon, Chul-Hwan Lee, Bhumsuk Keam, Se-Hoon Lee, Doo Hyun Chung, Yoon Kyung Jeon

**Affiliations:** 1 Cancer Research Institute, Seoul National University, Seoul, Republic of Korea; 2 Interdisciplinary Program of Cancer Biology, Seoul National University Graduate School, Seoul, Republic of Korea; 3 Department of Pathology, Seoul National University Hospital, Seoul National University College of Medicine, Seoul, Republic of Korea; 4 Department of Pathology, Korea University Guro Hospital, Korea University College of Medicine, Seoul, Republic of Korea; 5 Department of Biomedical Sciences, Seoul National University College of Medicine, Seoul, Republic of Korea; 6 Department of Pharmacology, Seoul National University College of Medicine, Seoul, Republic of Korea; 7 BK21 FOUR Biomedical Science Project, Seoul National University College of Medicine, Seoul, Republic of Korea; 8 Department of Internal Medicine, Seoul National University Hospital, Seoul National University College of Medicine, Seoul, Republic of Korea; 9 Division of Hematology-Oncology, Department of Medicine, Samsung Medical Center, Sungkyunkwan University School of Medicine, Seoul, Republic of Korea; 10 Department of Health Sciences and Technology, Samsung Advanced Institute of Health Sciences and Technology, Sungkyunkwan University, Seoul, Republic of Korea

**Keywords:** Non-small-cell lung cancer, Cancer microenvironment, Cancer immunotherapy, Predictive markers

Correction to: *British Journal of Cancer* 10.1038/s41416-024-02698-4, published online 10 May 2024

Following the publication of the article, the authors identified typographical errors within some of the correlation plots presented, stemming from having used the ‘slope value of graph’ rather than ‘coefficient Rho value’ from the statistical analyses results. The areas affected are:

- Fig 5G: CTL signature / High PD-L1
- Fig 6B: Vim H-score / PD
- Fig 6C: CD8 / PD (E-cad H-score), and CD8 / PD (Twist1 H-score)
- Supplemental Fig 11A: CD8 / CR+PR (E-cad H-score), and CD8 / CR+PR (Twist1 H-score)

There has been no change to the raw data presented; the changes do not impact the scientific interpretation of the relevant results.


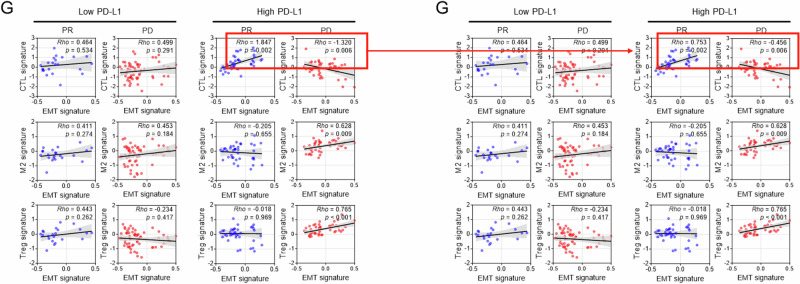



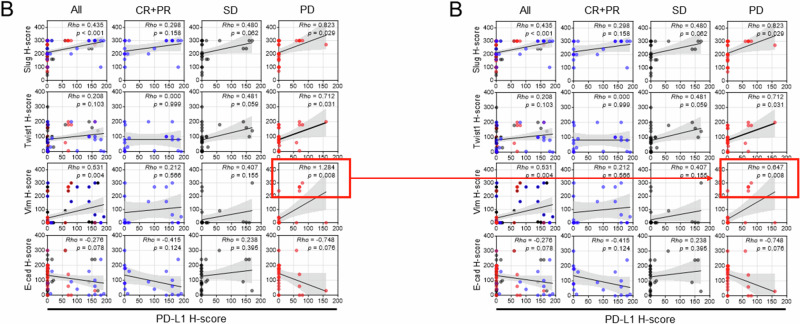



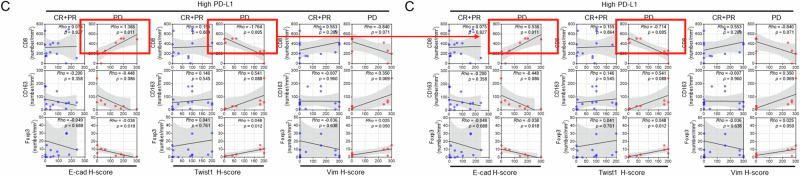



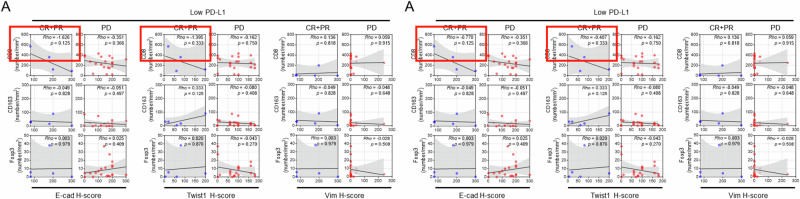


The original article has been corrected.

