# Supplementary material for: Epithelial−mesenchymal transition induced by tumor cell-intrinsic PD-L1 signaling predicts a poor response to immune checkpoint inhibitors in PD-L1-high lung cancer
Source: Br J Cancer. 2024 May 10;131(1):23–36. doi: 10.1038/s41416-024-02698-4 (PMC11231337; doi:10.1038/s41416-024-02698-4)
Supplement: Supplementary file 1 — Supplementary Figures [file 41416_2024_2698_MOESM1_ESM.docx]

**Supplementary Figures**

**
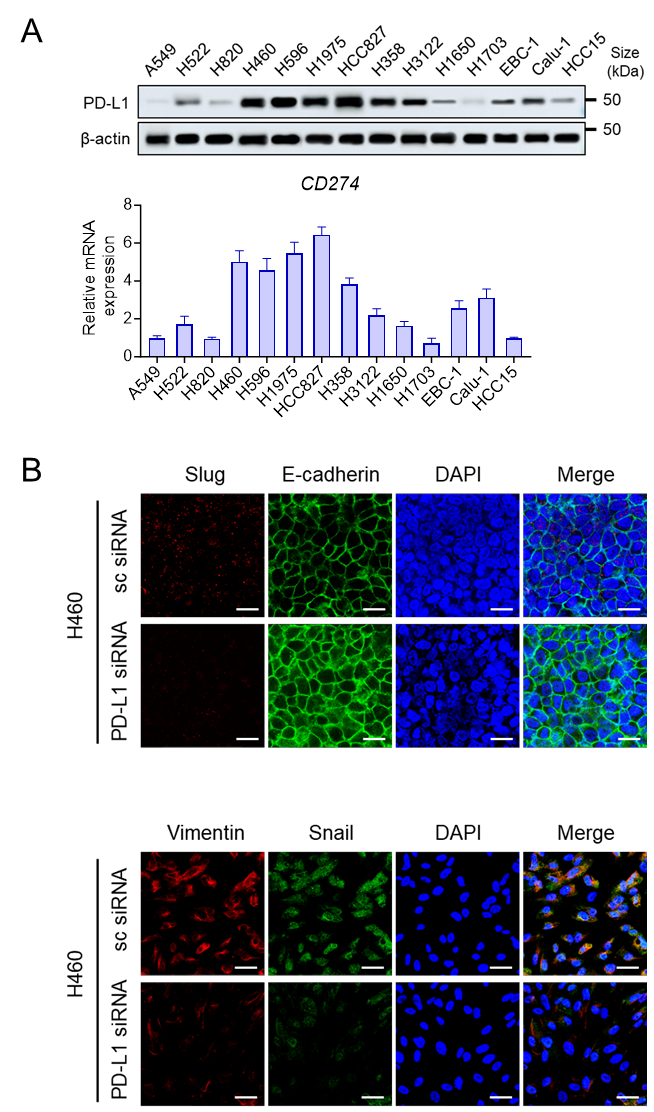
**

**Supplementary Figure S1. Expression of PD-L1 intrinsically promotes EMT in lung cancer cells**

(**A**) The basal expression of mRNA and protein of PD-L1 in human non-small cell lung cancer cell lines were measured by qRT-PCR and western blot, respectively. (**B**) H460 cells were transfected with PD-L1 siRNA or scrambled siRNA. At 48 h after transfection, protein expression of EMT markers was analyzed using immunofluorescence staining. Scale bar for E-cadherin = 10 μm. Scale bar for Snail = 20 μm.


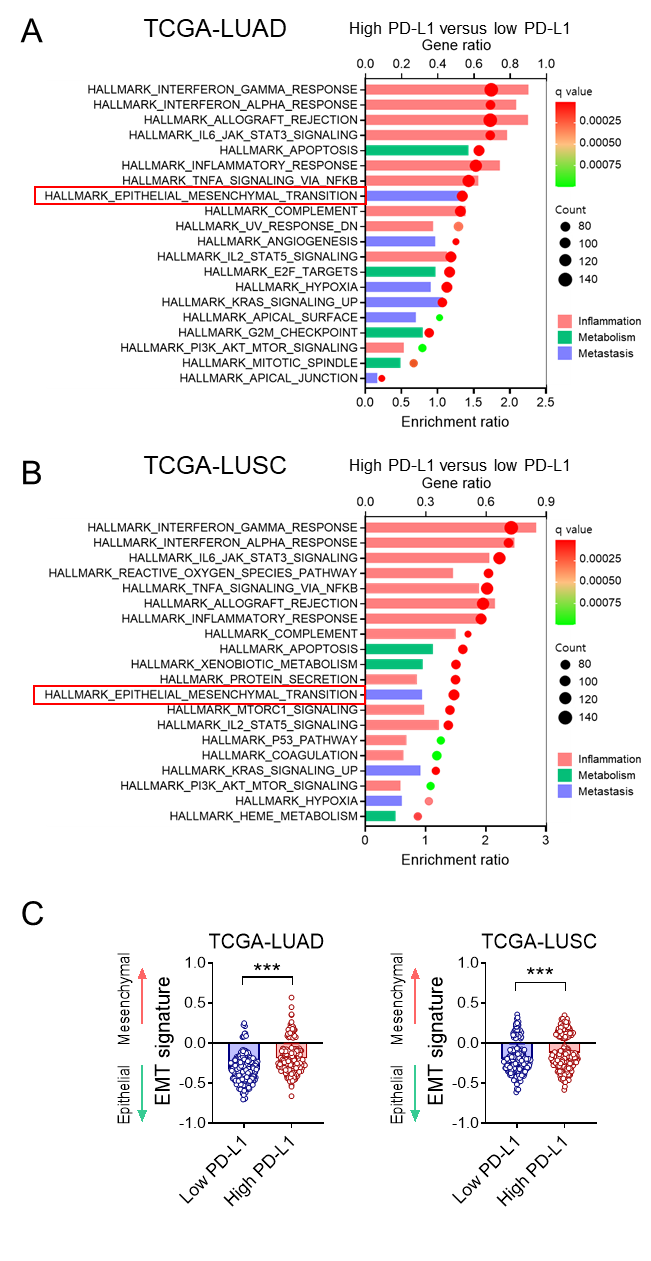
**Supplementary Figure S2. EMT pathway is enriched in PD-L1-high NSCLC (TCGA analysis)**

Hallmark gene set enrichment analysis with the (**A**) TCGA-LUAD (lung adenocarcinoma) and (**B**) TCGA-LUSC (lung squamous cell carcinoma) gene expression datasets. (**C**) EMT signature scores of TCGA-LUAD and TCGA-LUSC samples according to PD-L1 expression (low vs. high with a cutoff of median value). ****p* < 0.001.

**
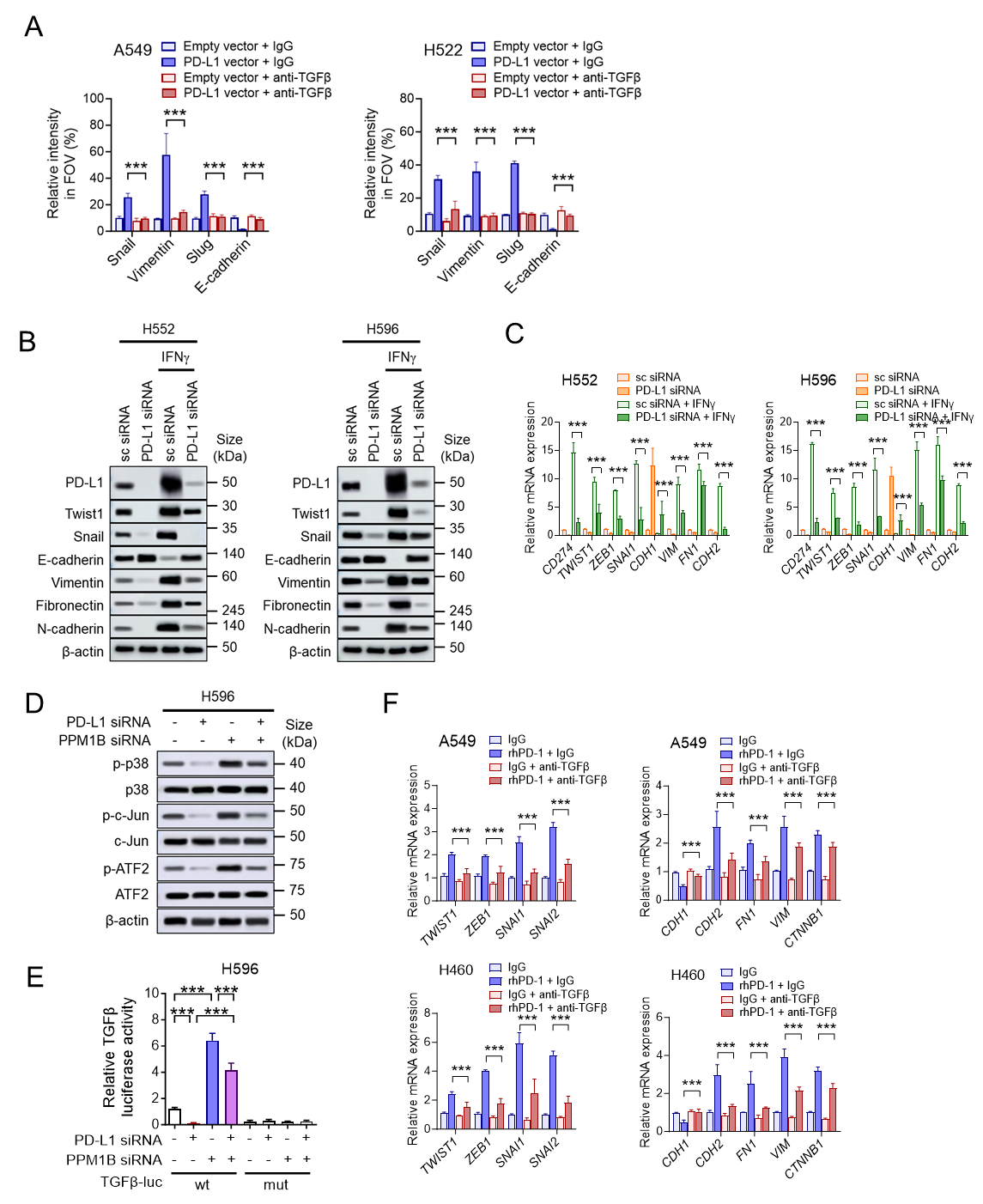
**

**Supplementary Figure S3. PD-L1 promotes EMT by** **via TGFβ production**

**(A)** A549 and H522 cells were transfected with PD-L1-expressing or empty vector in the presence or absence of anti-TGFβ neutralizing antibody (1 μg/mL). At 24 h after transfection, expression for EMT markers were analyzed by immunofluorescence staining and the fluorescence intensity in field of view (FOV) using CLSM Analysis Software LAS-X (Leica, Wetzlar, Germany).

H522 and H596 cells were treated with IFNγ with or without PD-L1 knockdown and protein and mRNA expression of EMT markers was analyzed by **(B)** Western blotting and **(C)** qRT-PCR. **(D)** H596 cells were transfected with PD-L1 siRNA and/or PPM1B siRNA. At 24 h after transfection, cell were submitted to Western blotting for indicated molecules. **(E)** H596 cells were transfected with PD-L1 siRNA and/or PPM1B siRNA for 24 h and then transfected with luciferase-expressing vector with promoter sequence of TGFβ containing ATF2- and c-Jun-binding motif. At 12 h after transfection, luciferase activity was measured. (**E**) A549 and H460 cells were treated with rhPD-1 (1 μg/ml) in the presence or absence of anti-TGFβ neutralizing antibodies. At 24 h after treatment, total RNA was extracted and submitted to qRT-PCR for EMT markers. Histograms represent values normalized to control. Data are presented as mean ± S.E.M. of three independent experiments. ****p* < 0.001.

**
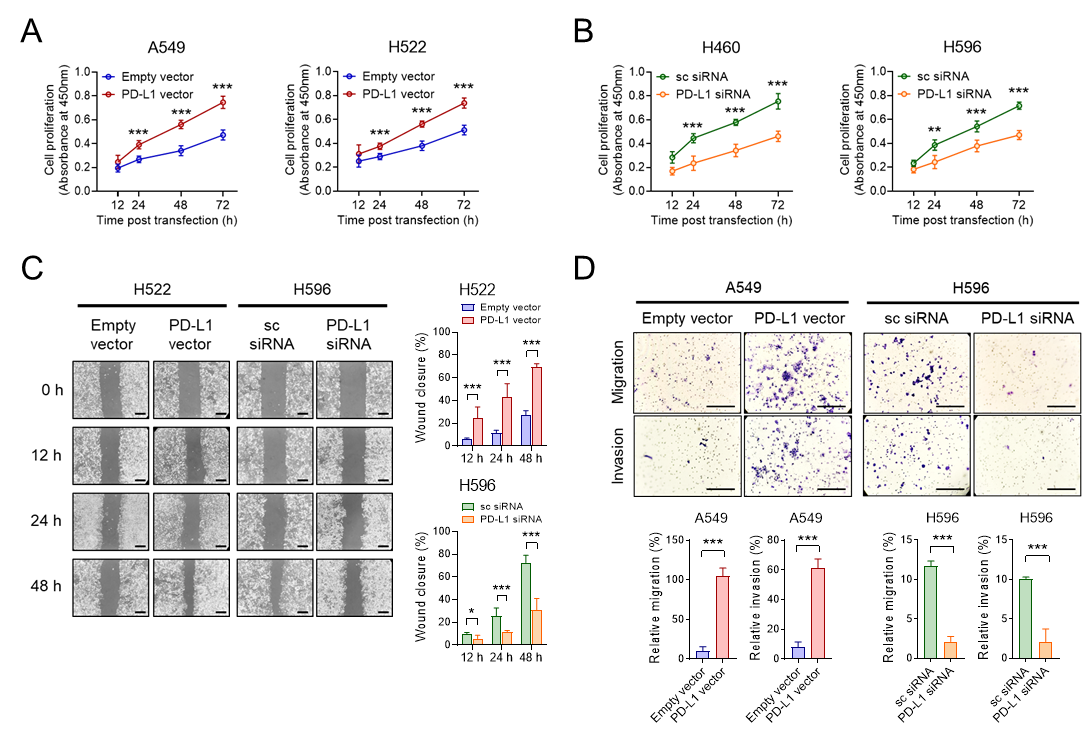
**

**Supplementary Figure S4. PD-L1 promotes proliferation, migration, and invasion of lung cancer cells**

A549 and H522 cells were transfected with PD-L1-expressing or empty vector, and H460 and H596 cells were transfected with PD-L1 siRNA or scrambled siRNA. Then the cells were submitted to **(A, B)** a cell proliferation assay using the CCK-8 assay and **(C)** a wound healing assay. The wound closure rate was calculated using ImageJ. Scale bar = 1,000 μm. **(D)** Cell migration and invasion assays were performed using Transwells, and the traversed cells after 24 h incubation were counted using ImageJ. Scale bar = 1,000 μm. Histograms represent values normalized to control. Data are presented as mean ± S.E.M. of three independent experiments. **p* < 0.05, ***p* < 0.01, and ****p* < 0.001.

**
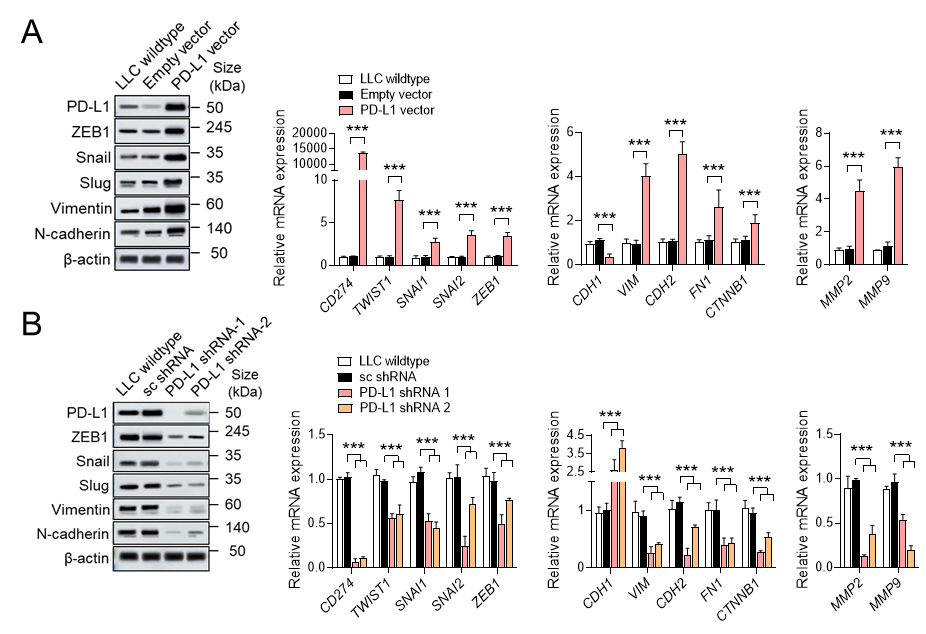
**

**Supplementary Figure S5. PD-L1 promotes EMT in mouse cancer cells**

Mouse lung cancer cells LLC were infected with lentivirus using pLVX-mCherry-mPD-L1 or pLKO.1-mCherry-mPD-L1 shRNAs. (**A, B**) Protein and mRNA expression of EMT markers was analyzed using Western blotting and qRT-PCR. Histograms represent values normalized to control. Data are presented as mean ± S.E.M. of three independent experiments. **p* < 0.05, ***p* < 0.01, and ****p* < 0.001.


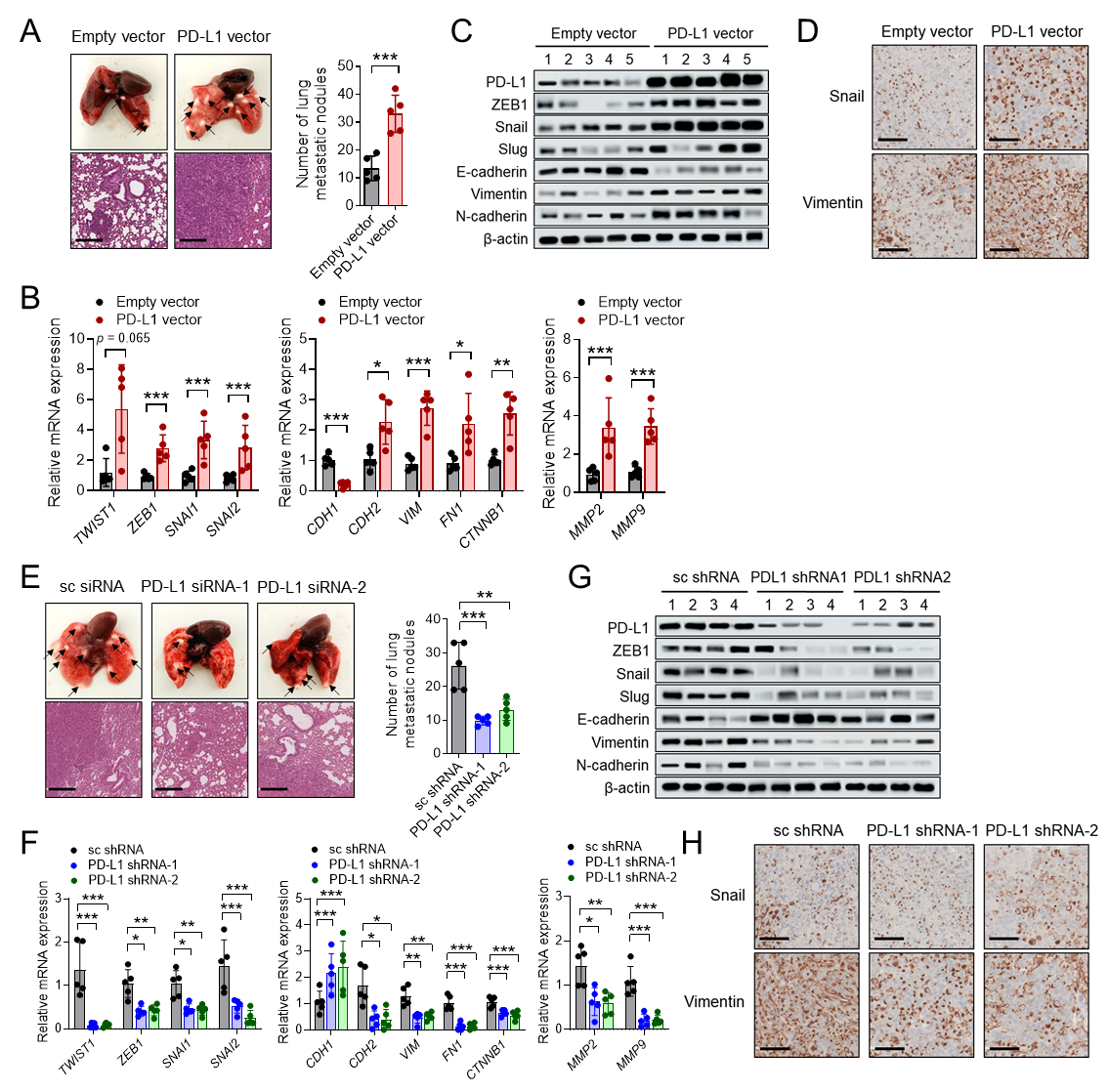


**Supplementary Figure S6. PD-L1 enhances pulmonary metastasis in tail vein injection model**

PD-L1-overexpressing or vector control LLC cells and PD-L1-knockdown or shRNA control LLC cells were injected into Balb/c-nu mice via the tail vein. **(A, E)** Lung metastases nodules were counted at dpi 25. Representative H&E stained images of lung showing metastatic lesions. **(B, C, F, G)** The expression of EMT markers in lung was analyzed by qRT-PCR and Western blotting. **(D, H**) Representative IHC staining images for Snail and Vimentin of lung tumors. Data in histograms are presented as mean ± S.E.M. of three independent experiments. **p* < 0.05, ***p* < 0.01, and ****p* < 0.001.

***
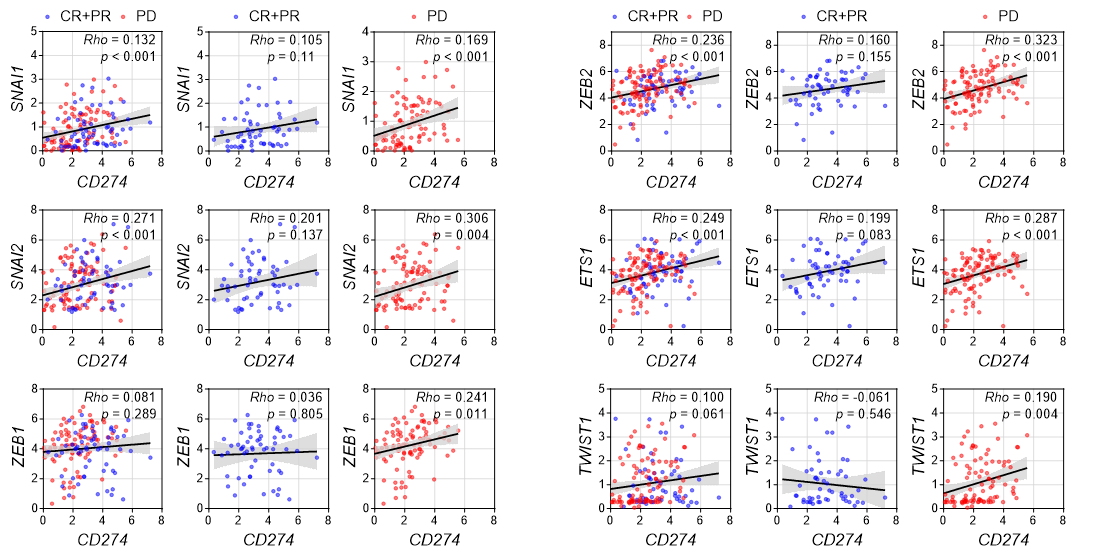
***

**Supplementary Figure S7. PD-L1 expression is significantly correlated with EMT transcription factor expression in patients with NSCLC showing PD after ICI therapy**

Correlation between PD-L1 expression and EMT transcription factor expression in patients showing partial response (PR, blue dot) and progressive disease (PD, red dot). Correlations among variables were calculated using Spearman’s correlation test.

**
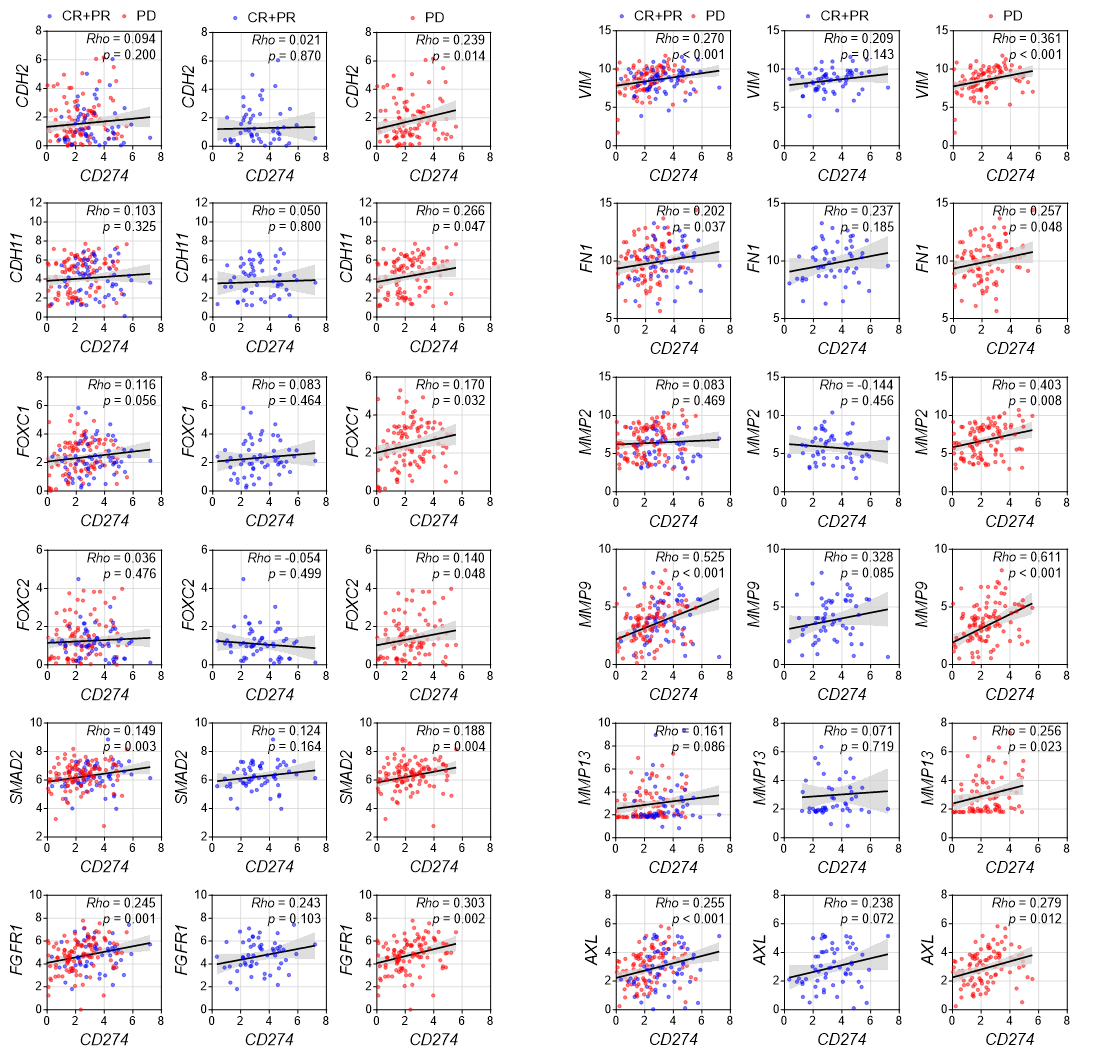
Supplementary Figure S8*.* PD-L1 expression is significantly correlated with mesenchymal expression in patients with NSCLC showing PD after ICI therapy**

Correlation between PD-L1 expression and mesenchymal marker expression in patients showing partial response (PR, blue dot) and progressive disease (PD, red dot). Correlations among variables were calculated using Spearman’s correlation test.


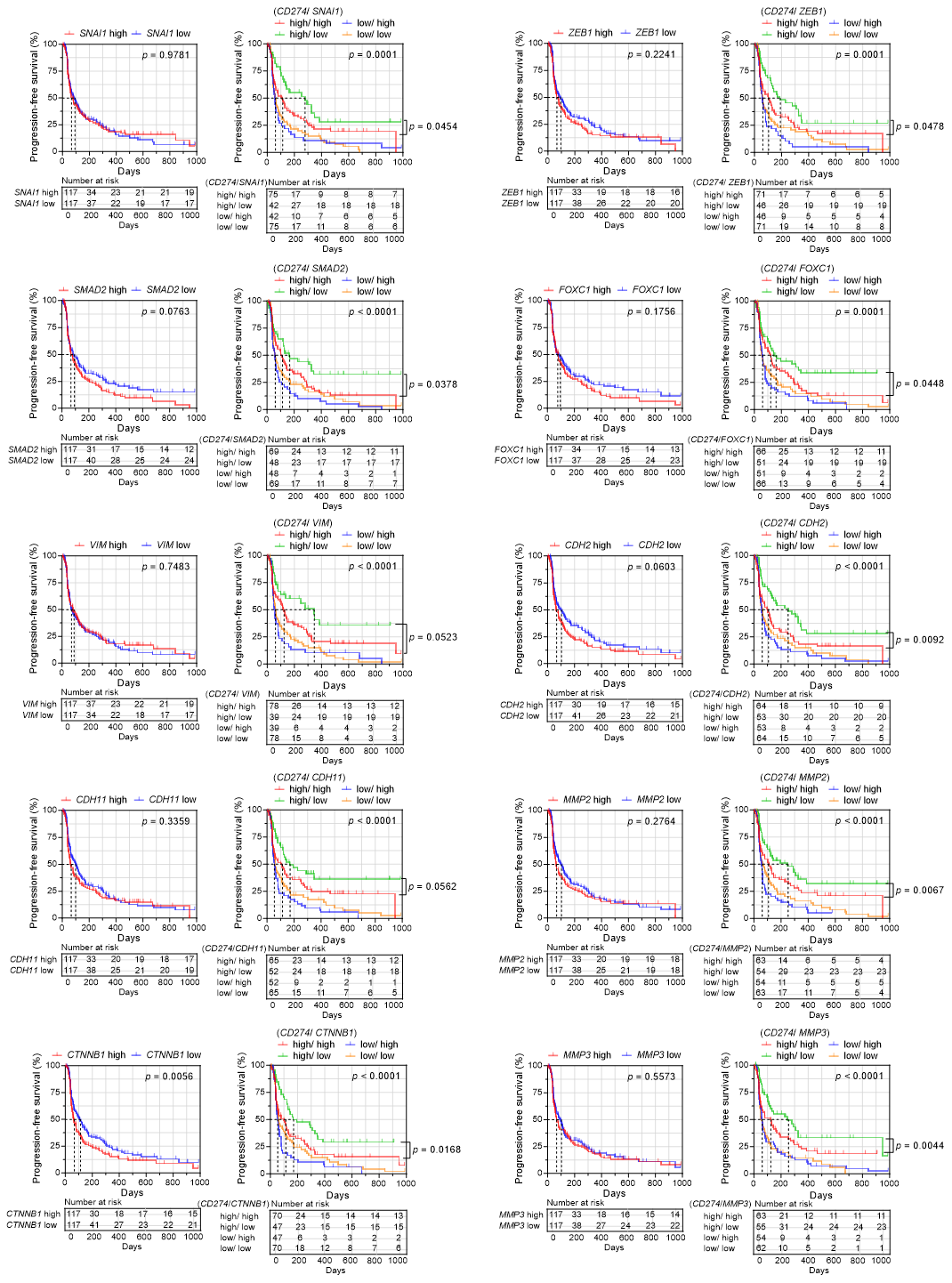


**Supplementary Figure S9. Higher mesenchymal marker expression is associated with poor clinical outcomes after ICI therapy in patients with PD-L1-high NSCLC**

All patients from the RNA-seq ICI cohort were divided into low versus high groups in terms of PD-L1 expression and each mesenchymal marker expression according to median values. Differences in PFS were analyzed between the groups using Kaplan–Meier analysis with the log-rank test.

**
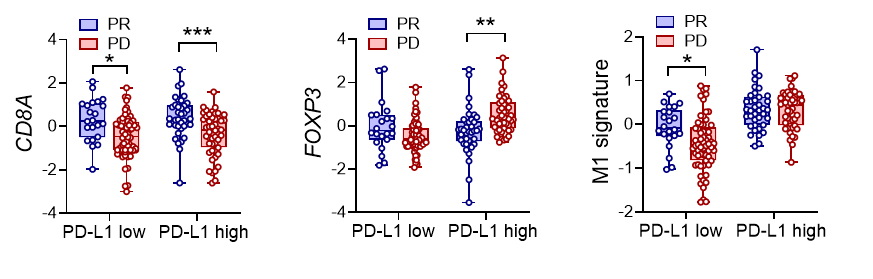
**

**Supplementary Figure S10. Immunosuppressive TME in patients with PD after ICI therapy in PD-L1-high NSCLC (RNA-seq cohort)**

Differences in *CD8A*, *FOXP3*, and M1 signature expression between patients showing PR and PD to ICI therapy in PD-L1-low and PD-L1-high NSCLC groups, respectively. Data in histograms are presented as mean ± S.E.M. of three independent experiments. **p* < 0.05, ***p* < 0.01, and ****p* < 0.001.


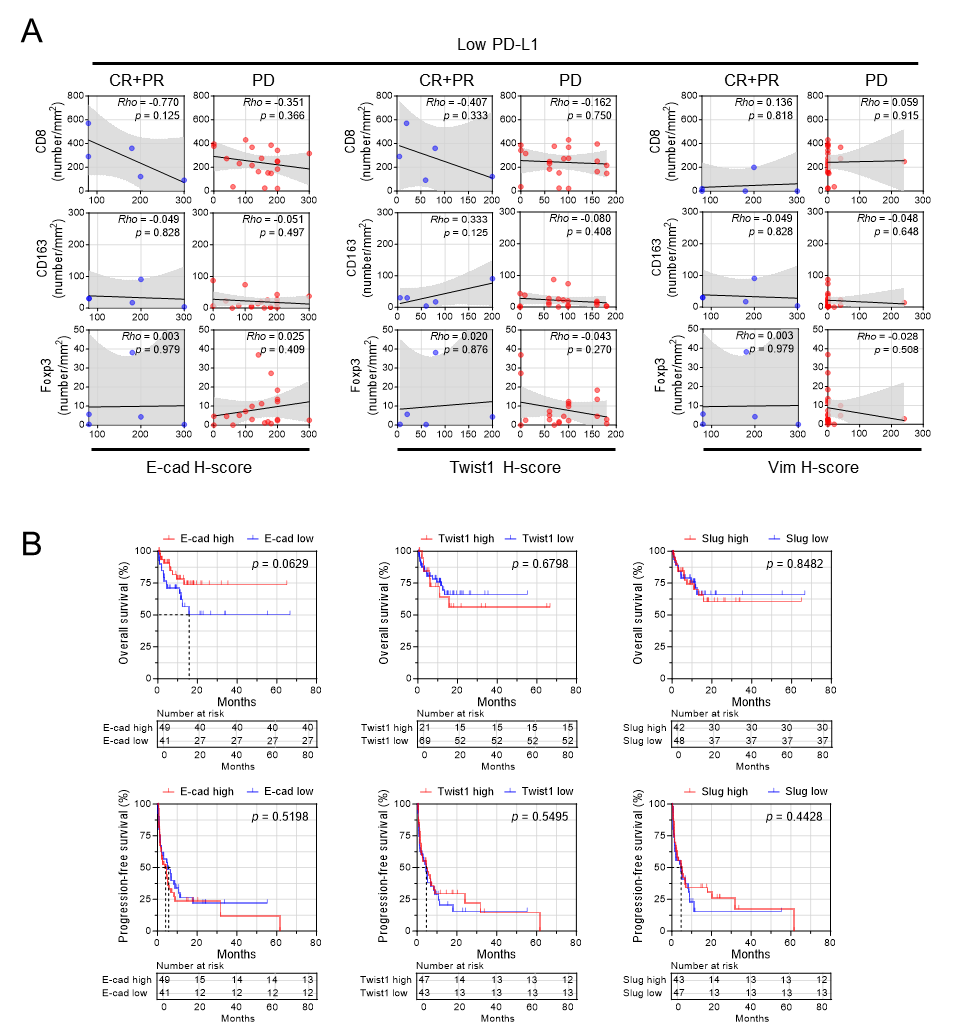


**Supplementary Figure S11. Limited role of EMT in tumor cells as a biomarker for poor clinical outcomes after ICI therapy in patients with PD-L1-low NSCLC (IHC cohort)**

(**A**) Correlations between the H-score of EMT molecules and the numbers of CD8^+^, CD163^+^, and Foxp3^+^ cells in patients showing a complete response plus PR and PD, respectively, in PD-L1-low NSCLCs. (**B**) Kaplan−Meier analysis of overall survival and PFS after ICI therapy according to EMT molecule expression. The survival difference was analyzed using the Kaplan–Meier method and log-rank test. Correlations among variables were calculated using Spearman’s correlation test.


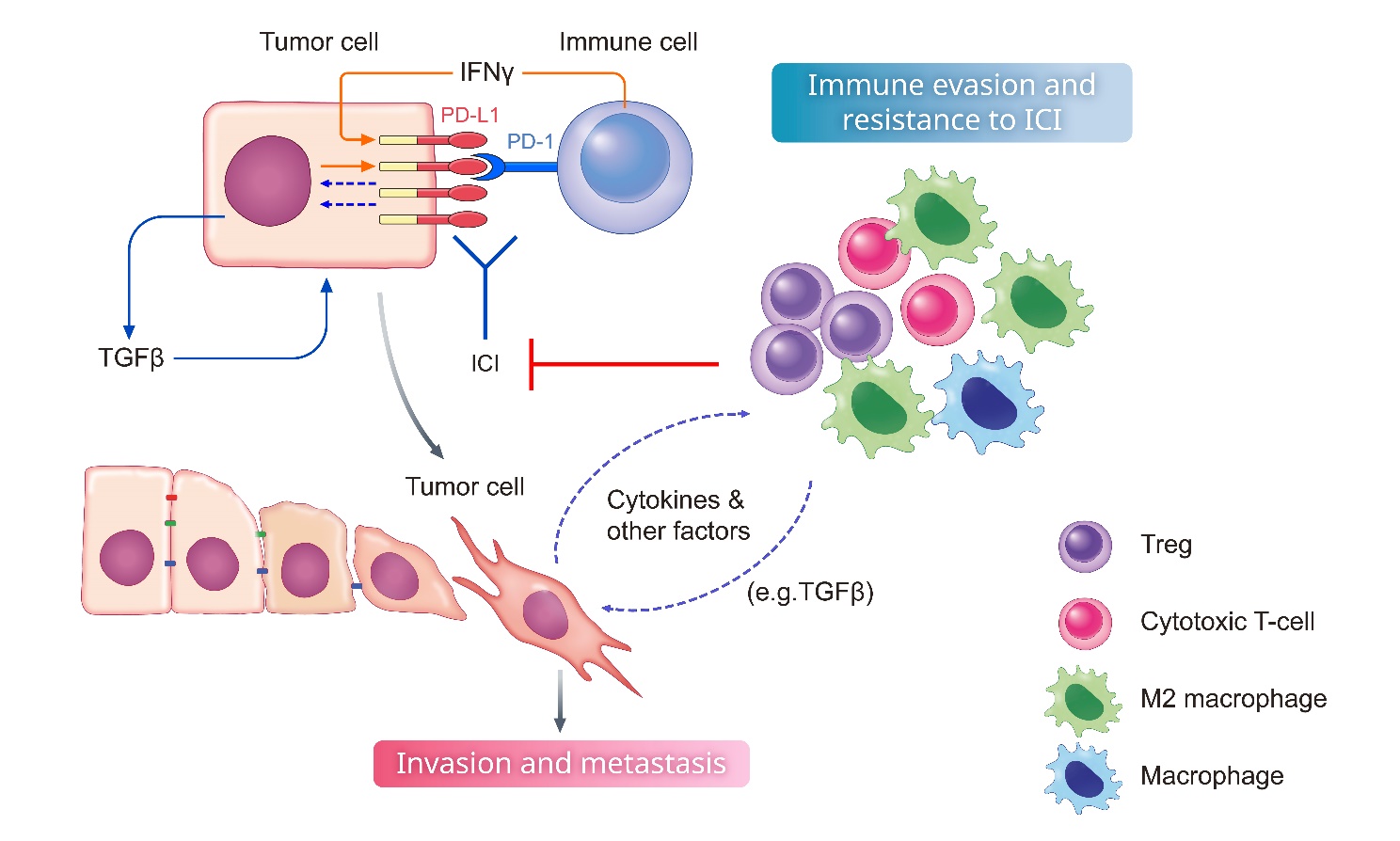


**Supplementary Figure S12. Working model**

Tumor cell-intrinsic PD-L1 function induces EMT by TGFβ and promotes progression and metastasis of NSCLC *in vitro* and *in vivo* and that the predictive role of EMT in ICI therapy in patients with NSCLC depends on the PD-L1 expression status. EMT is associated with increased M2 macrophage and Treg infiltrations and decreased CTL infiltration and predicts an unfavorable response and outcome after ICI therapy in PD-L1-high NSCLC.
