# Supplementary material for: Epithelial−mesenchymal transition induced by tumor cell-intrinsic PD-L1 signaling predicts a poor response to immune checkpoint inhibitors in PD-L1-high lung cancer
Source: Br J Cancer. 2024 May 10;131(1):23–36. doi: 10.1038/s41416-024-02698-4 (PMC11231337; doi:10.1038/s41416-024-02698-4)
Supplement: Supplementary file 2 — Supplementary Methods and Tables [file 41416_2024_2698_MOESM2_ESM.docx]

**Supplementary Information**

**Supplementary Methods**

**RNA extraction and qRT-PCR**

Total mRNA was extracted from cells and tumor tissues using TRIzol reagent (Molecular Research Center), quantified, and transcribed into complementary DNA by reverse transcriptase (RR036A; Takara Bio Inc., Shiga, Japan). The transcription level of target genes was detected using a CFX Connect Real-Time PCR System (Bio-Rad, Hercules, CA, USA) and analyzed by qRT-PCR with sets of primers (**Supplementary Table S1**). The housekeeping gene β-actin was used an internal control. The fold change was calculated using the delta-delta C_T_ method and normalized by the averaged delta C_T_ value of the negative control group.

**Co-immunoprecipitation and Western blot**

For co-immunoprecipitation, HEK293T cells were collected and lysed on ice for 30 min. The cell lysates were incubated with indicated antibodies at 4℃ overnight, followed by incubating with Protein A/G Plus-Agarose (sc-2003, Santa Cruz) at 4℃ for another 2 h. The immunocomplex was washed 4 times and boiled in 5X SDS sample buffer for 5 min. The co-precipitates were resolved using SDS-PAGE and subjected to immunoblotting.

For immunoblotting, cells were lysed with RIPA buffer (Thermo Fisher Scientific, Waltham, MA, USA) containing a protease (GenDEPOT, Baker, TX, USA) and phosphatase inhibitor cocktail (P3200; GenDEPOT). The protein concentration of each cell lysate was quantified using a protein quantification kit (Biomax, Houston, TX, USA), boiled for 5 min at 95℃, loaded onto 8% to 10% SDS-polyacrylamide gel, and transferred onto a polyvinylidene fluoride membrane (Millipore, Burlington, MA, USA). Then the membranes were blocked in 5% bovine serum albumin in Tris-buffered saline with Tween-20 for 1 h at room temperature and incubated with primary antibodies at 4℃ overnight, followed by incubation with horseradish peroxidase-conjugated secondary antibodies for 2 h at room temperature. The protein blots were detected using enhanced chemiluminescence reagents (ATTO, Tokyo, Japan). The antibodies used for immunoblotting are listed in **Supplementary Table S2**.

**CCK-8 assay**

Cells were seeded into 96-well plates. At 24 h after transfection of PD-L1-expressing vector or PD-L1 siRNAs, 10 μL CCK-8 solution (D-Plus cell viability assay reagent) (CCK-3000; Dongin Biotech, Seoul, Republic of Korea) was added into each well, and then the wells were incubated at 37℃ for 5 min in the dark. The optical density at 450 nm was detected by a microplate reader.

**Wound-healing assay**

Cells were seeded into 12-well plates and transfected with a PD-L1-expressing vector or PD-L1 siRNA. Wounds were introduced to a monolayer of cells using a sterile 200 μL pipette tip 24 h after transfection. Then, the wounded monolayer of cells was washed with sterile PBS and cultured. The speed of wound closure was observed and calculated using ImageJ.

**Migration and invasion assay**

Cell migration and invasion were detected using 8 μm pore Transwells (37224; SPL Life Sciences, Gyeonggi-do, Republic of Korea) and 8 μm pore Transwells coated with Matrigel (354480; Corning Inc., Corning, NY, USA), respectively. Cells were transfected with PD-L1-expressing vector or PD-L1 siRNA, and seeded in the upper well of the Transwell chamber. The lower compartment was filled with media containing 20% FBS. After 24 h of incubation, the cells in the upper well were wiped using a wet cotton swab, and traversed cells on the lower side of the filter were fixed and stained with crystal violet. The cells were counted microscopically and quantified by ImageJ.

**Immunofluorescence staining**

The cells were plated on glass coverslips. After transfection with a PD-L1-expressing vector or PD-L1 siRNA, the cells were fixed in 4% paraformaldehyde, permeabilized in 0.1% Triton X-100 in PBS, and then blocked with 2% bovine serum albumin in PBS for 1 h. Then the cells were incubated with primary antibodies at 4℃ overnight. The cells were washed three times and subsequently incubated with FITC-conjugated secondary antibody (A11001; Invitrogen, Waltham, MA, USA) and Alexa Fluor 647-conjugated secondary antibody (A21246; Invitrogen) in a dark room for 1 h. Nuclei were stained with DAPI for 5 min at room temperature. Fluorescence intensity was observed using a confocal laser scanning microscope (Leica TCS SP8; Leica, Wetzlar, Germany).

***In vitro* phosphatase assay**

Phosphatase reactions were conducted by incubating recombinant human p-p38 protein (ab271647; Abcam, Cambridge, UK) as a substrate with recombinant human PPM1B (LS-G21072-20; LS Bio, Lynnwood, WA, USA) as a phosphatase in phosphatase reaction buffer (25 mM HEPES pH7.4, 150 mM NaCl, 0.1 mM EDTA, 2.5 mM DTT, 1.5 mM BSA, 0.02% Brij 35) at 30℃ for 30 min in the presence or absence of recombinant human PD-L1 (156-B7; R&D Systems). Sanguinarine chloride (PHL89327; Sigma-Aldrich) was used as a specific inhibitor of protein phosphatase. To estimate phosphorylation status of p38, immunoblotting using antibodies against p-p38 and measurement of free phosphate were performed. Free phosphate were released from phosphorylated proteins due to enzyme activity of phosphatase in reaction buffer, which was treated with reagents of Malachite Green Phosphate Detection kit (DY996; R&D Systems) and measured using an enzyme-linked immunosorbent assay reader (Bio Tek, Winooski, VT, USA) at 620 nm.

**Pull-down assay**

In-Fusion technology provided from Takara Bio was used for deletion mutagenesis of human PD-L1 (*CD274*). The sequence of extracellular domain (ECD), transmembrane domain (TMD), and intracellular domain (ICD) in PD-L1 were searched using the NetNES1.1 Server program (<http://www.cbs.dtu.dk/services/NetNES/>). After determining the sequence of ECD, TMD, and ICD of *CD274*, primers were listed in **Supplementary Table S6**. Polymerase chain reaction was performed with the template pGEX-human PD-L1 plasmid (121466; Addgene, Watertown, MA, USA) at 95°C for 1 min for denaturation, 55°C for 1 min for annealing, and 72°C for 7 min for elongation, for total 35 cycles. Thereafter, 20 units of Dpn1 (R054M; Enzynomics, Republic of Korea) were added, followed by transformation using DH5α competent *E. coli*. Plasmid DNA were extracted using EZ-pure plasmid prep kit (EP101-200N; Enzynomics), and the deletion was confirmed by Bioneer (Republic of Korea). Then, GST-fused PD-L1 (amino acids 1-238, 259-290, and full length) were expressed in *E. coli* and purified using glutathione resin in Pierce GST spin purification kit (16106; Thermo Fisher Scientific). His-PPM1B protein (LS-G21072-20) were purchased from LS Bio (Seattle, WA, USA). For GST pull-down assay, 1 μg of GST, GST-PD-L1 full length (FL), GST-PD-L1 ECD, or GST-PD-LE ICD was incubated with 1 μg of His or His-tagged PPM1B, together with Glutathione Sepharose 4 Fast Flow in PBS with 1% Triton X-100 for 3 h at 4℃. The beads were washed and each purified protein was analyzed by western blot.

**Luciferase reporter assay**

To construct a reporter plasmid, a 129-bp region of the human TGFβ promoter that includes ATF2 (TGA-GTCA)- and c-Jun-binding motif (TGA-GTCA) was cloned into the luciferase-expressing plasmid pGL4.24-luc2P (E842A, Promega). Single strand oligos were designed to contain the part of TGFβ promoter sequence (listed in **Supplementary Table S6**). After annealing, the inserts were incubated with the restriction enzymes KpnI and NheI, and ligated with the luciferase-expressing plasmid. Luciferase assay was performed using Dual-luciferase Reporter Assay System (E1910, Promega, WI, USA). Cells were seeded in 6-well plate and transfected with PD-L1 siRNA or PPM1B siRNA for 24 h. After washed with PBS three times, the cells were transfected with luciferase-expressing plasmid pGL4.24-luc2P-hTGFβ. After 12 h, the cells were lysed and subjected to luciferase activity measurement according to manufacturer’s instructions. As a negative control, the plasmid with truncated mutation of the TGFβ promoter sequence were used.

**Immunohistochemistry (IHC)**

IHC for Slug, Twist1, vimentin, E-cadherin, CD8, CD163, and FOXP3 was performed using the antibodies listed in **Supplementary Table S2** and BenchMark XT autostainer (Ventana Medical Systems, Tucson, AZ, USA). PD-L1 IHC was performed using a PD-L1 IHC 22C3 pharmDx system (Agilent Technologies). Whole-slide images were obtained using an Aperio ScanScope slide scanner (Aperio Technologies, Vista, CA, USA). Protein expression was evaluated by the H-score, which ranged from 0 to 300 and was calculated using the following formula: H-score = (3 × percentage of strongly staining cells) + (2 × percentage of moderately staining cells) + (1 × percentage of weakly staining cells). For PD-L1, the tumor proportion score (TPS) was also estimated. The number/mm^2^ of CD8^+^, CD163^+^, and FOXP3^+^ cells was automatically counted using QuPath software.

**Supplementary Tables**

**Supplementary Table S1.** Primers for quantitative real-time polymerase chain reaction

| **Target gene** | **Species** | **Strand** | **Sequence (5′→3′)** |
| --- | --- | --- | --- |
| PD-L1 (*CD274*) | Human | Forward | TAT GGT GGT GCC GAC TAC AA |
|  |  | Reverse | TGC TTG TCC AGA TGA CTT CG |
| *TWIST1* | Human | Forward | TGC ATG CAT TCT CAA GAG GT |
|  |  | Reverse | GTT TTG CAG GCC AGT TTG AT |
| Snail (*SNAI1*) | Human | Forward | GCT CCA CAA GCA CCA AGA GT |
|  |  | Reverse | ATT CCA TGG CAG TGA GAA GG |
| Slug (*SNAI2*) | Human | Forward | CTT TTT CTT GCC CTC ACT GC |
|  |  | Reverse | ACA GCA GCC AGA TTC CTC AT |
| Vimentin (*VIM*) | Human | Forward | GAG AAC TTT GCC GTT GAA GC |
|  |  | Reverse | TCC AGC AGC TTC CTG TAG GT |
| Fibronectin (*FN1*) | Human | Forward | ACC AAC CTA CGG ATG ACT CG |
|  |  | Reverse | GCT CAT CAT CTG GCC ATT TT |
| E-cadherin (*CDH1*) | Human | Forward | CAA TGC CGC CAT CGC TTA C |
|  |  | Reverse | ATG ACT CCT GTG TTC CTG TTA ATG |
| N-cadherin (*CDH2*) | Human | Forward | GAC AAT GCC CCT CAA GTG TT |
|  |  | Reverse | CCA TTA AGC CGA GTG ATG GT |
| β-catenin (*CTNNB1*) | Human | Forward | CAA CTA AAC AGG AAG GGA TG |
|  |  | Reverse | CAC AGG TGA CCA CAT TTA TAT C |
| *MMP2* | Human | Forward | AGA AGG CTG TGT TCT TTG CAG |
|  |  | Reverse | AGG CTG GTC AGT GGC TTG |
| *MMP9* | Human | Forward | GAA CCA ATC TCA CCG ACG GG |
|  |  | Reverse | GCC ACC CGA GTG TAA CCA TA |
| *ZEB1* | Human | Forward | CGT TTC TTG CAG TTT GGG CAT T |
|  |  | Reverse | AAG AAT TCA CAG TGG AGA GAA GCC A |
| β-actin | Human | Forward | AGG CCA CCC CAG AGG ACA AC |
|  |  | Reverse | CCA GAG GCG TAC AGG GAT A |
| PD-L1 (*CD274*) | Mouse | Forward | CTC GCC TGC AGA TAG TTC CC |
|  |  | Reverse | GTC CAG CTC CCG TTC TAC AG |
| *ZEB1* | Mouse | Forward | ACA AGA CAC CGC CGT CAT TT |
|  |  | Reverse | GCA GGT GAG CAA CTG GGA AA |
| *TWIST1* | Mouse | Forward | CGG GTC ATG GCT AAC GTG |
|  |  | Reverse | CAG CTT GCC ATC TTG GAG TC |
| Snail (*SNAI1*) | Mouse | Forward | CCA CTG CAA CCG TGC TTT T |
|  |  | Reverse | CAC ATC CGA GTG GGT TTG G |
| Slug (*SNAI2*) | Mouse | Forward | CTC ACC TCG GGA GCA TAC AGC |
|  |  | Reverse | TGA AGT GTC AGA GGA AGG CGG G |
| Vimentin (*VIM*) | Mouse | Forward | TTC TCT GGC ACG TCT TGA CC |
|  |  | Reverse | CTC CTG GAG GTT CTT GGC AG |
| Fibronectin (*FN1*) | Mouse | Forward | ATG ACG ATG GGA AGA CCT AC |
|  |  | Reverse | GGC TGG AAA GAT TAC TCT CG |
| E-cadherin (*CDH1*) | Mouse | Forward | GGT TTT CTA CAG CAT CAC CG |
|  |  | Reverse | GCT TCC CCA TTT GAT GAC AC |
| N-cadherin (*CDH2*) | Mouse | Forward | TGA AAC GGC GGG ATA AAG AG |
|  |  | Reverse | GGC TCC ACA GTA TCT GGT TG |
| β-catenin (*CTNNB1*) | Mouse | Forward | ATT GAT TCG AAA CCT TGC CC |
|  |  | Reverse | AGC TCC AGT ACA CCC TTC TA |
| *MMP2* | Mouse | Forward | GAT AAC CTG GAT GCC GTC GTG |
|  |  | Reverse | CTT CAC GCT CTT GAG ACT TTG GTT C |
| *MMP9* | Mouse | Forward | GCC CTG GAA CTC ACA CGA CA |
|  |  | Reverse | TTG GAA ACT CAC ACG CCA GAA G |
| β-actin | Mouse | Forward | TGT CCA CCT TCC AGC AGA TGT |
|  |  | Reverse | AGC TCA GTA ACA GTC CGC CTA GA |

**Supplementary Table S2.** Antibodies used for immunoblotting, immunofluorescence staining, and immunohistochemistry

| **Antibody** | **Species** | **Clone** | **Company** | **Catalog no.** | **Application** |
| --- | --- | --- | --- | --- | --- |
| PD-L1 | Human | E1L3N | Cell Signaling Technology | 13684 | 1:1000 |
| Vimentin | Human, mouse | D21H3 | Cell Signaling Technology | 5741 | 1:1000 |
| E-cadherin | Human | Polyclonal | Santa Cruz Biotechnology | sc-7870 | 1:1000 |
| E-cadherin | Human, mouse | 24E10 | Cell Signaling Technology | 3195 | 1:1000 |
| N-cadherin | Human, mouse | D4R1H | Cell Signaling Technology | 13116 | 1:1000 |
| Fibronectin/FN1 | Human | E5H6X | Cell Signaling Technology | 26836 | 1:1000 |
| ZEB1 | Human, mouse | E2G6Y | Cell Signaling Technology | 70512 | 1:1000 |
| TWIST1 | Human | E7E2G | Cell Signaling Technology | 69366 | 1:1000 |
| Snail | Human, mouse | C15D3 | Cell Signaling Technology | 3879 | 1:1000 |
| Slug | Human, mouse | C19G7 | Cell Signaling Technology | 9585 | 1:1000 |
| β-actin | Human, mouse | Polyclonal | Bioworld Technology | AP0060 | 1:5000 |
| PD-L1 | Mouse | Polyclonal | R&D Systems | AF1019 | 1:1000 |
| PPM1B | Human | W19264A | Biolegend | 934201 | 1:1000 |
| Flag | All | D6W5B | Cell Signaling Technology | 14793 | 1:1000 |
| His | All | D3I1O | Cell Signaling Technology | 12698 | 1:1000 |
| p-p38 | Human,  mouse | D3F9 | Cell Signaling Technology | 4511 | 1:1000 |
| p38 | Human,  mouse | D13E1 | Cell Signaling Technology | 8690 | 1:1000 |
| p-ATF2 | Human,  mouse | E4A5G | Cell Signaling Technology | 27934 | 1:1000 |
| ATF2 | Human,  mouse | D4L2X | Cell Signaling Technology | 35031 | 1:1000 |
| p-c-Jun | Human,  mouse | D47G9 | Cell Signaling Technology | 3270 | 1:1000 |
| c-Jun | Human,  mouse | 60A8 | Cell Signaling Technology | 9165 | 1:1000 |
| Goat anti-rabbit IgG (HRP conjugate) | Rabbit | Polyclonal | Enzo Life Sciences | ADI-SAB-300-J | 1:10,000 |
| Goat anti-mouse IgG (HRP conjugate) | Mouse | Polyclonal | BioLegend | 405306 | 1:10,000 |
| Donkey anti-goat IgG (HRP conjugate) | Goat | Polyclonal | GeneTex | GTX232040-01 | 1:10,000 |
| Goat anti-mouse IgG (Alexa Fluor 488) | Mouse | Polyclonal | Thermo Fisher Scientific | A-11001 | 1:5000 |
| Goat anti-rabbit IgG (Alexa Fluor 647) | Rabbit | Polyclonal | Thermo Fisher Scientific | A-21246 | 1:5000 |
| PD-L1 for IHC | Human | 22C3 | Dako | PharmDx kit | PharmDx kit |
| CD8 for IHC | Human | SP16 | Thermo Fisher Scientific |  | 1:100 |
| CD163 for IHC | Human | OTI2G12 | Abcam |  | 1:200 |
| Foxp3 for IHC | Human | 236A/E7 | Abcam |  | 1:100 |
| Slug for IHC | Human | OTI1G7 | OriGene | TA800196 | 1:150 |
| Twist1 for IHC | Human | 3E1 | Invitrogen | MA5−32927 | 1:100 |
| Vimentin for IHC | Human,  mouse | D21H3 | Cell Signaling Technology | 5741 | 1:100 |
| E-cadherin for IHC | Human,  mouse | 24E10 | Cell Signaling Technology | 3195 | 1:400 |

IHC, immunohistochemistry.

**Supplementary Table S3.** Characteristics of patients in the RNA-seq ICI cohort

| Characteristics |  |  |
| --- | --- | --- |
| Age | Years | 59.9 (29.5−84.5) |
| Sex | Male | 168 (71.8) |
|  | Female | 66 (28.2) |
| ECOG PS | 0−1 | 206 (88.0) |
|  | 2 | 28 (12.0) |
| Smoking | Never | 77 (33.3) |
|  | Ever | 156 (66.7) |
| Histology | ADC | 155 (66.2) |
|  | SqCC | 69 (29.5) |
|  | Other* | 10 (4.3) |
| Genetic changes | *EGFR* mutation | 40 (17.3) |
|  | *KRAS* mutation | 18 (7.7) |
|  | *ALK* translocation | 8 (3.4) |
| Prior lines of chemotherapy | 0 | 15 (6.4) |
|  | 1 | 94 (40.2) |
|  | ≥2 | 125 (53.4) |
| Response to ICI therapy | Complete response | 0 (0.0) |
|  | Partial response | 62 (26.5) |
|  | Stable disease | 56 (23.9) |
|  | Progressive disease | 116 (49.6) |

Data are presented as median (range) or n (%). ECOG PS, Eastern Cooperative Oncology Group performance status; ADC, adenocarcinoma; SqCC, squamous cell carcinoma; ICI, immune checkpoint inhibitor

**Supplementary Table S4.** Characteristics of patients in the immunohistochemistry cohort

| Characteristics |  |  |
| --- | --- | --- |
| Age | Years | 63.9 (33−88) |
| Sex | Male  Female | 72 (80.0)  18 (20.0) |
| ECOG PS | 0−1  2 | 88 (97.8)  2 (2.2) |
| Smoking | Never  Ever | 27 (30.0)  63 (70.0) |
| Histology | ADC  SqCC  Other^a^ | 48 (53.3)  28 (31.1)  14 (15.6) |
| Tumor size | cm | 3.7 (0.6−11.5) |
| LN metastasis^*^ | N0 + N1  N2 | 58 (64.4)  27 (30.0) |
| Stage at diagnosis | I  II  III−IV | 23 (25.6)  22 (24.4)  45 (50.0) |
| Immunotherapy | Anti-PD-L1^b^  Anti-PD-1^c^ | 25 (27.8)  65 (72.2) |
| Response to ICI therapy^*^ | Complete response  Partial response  Stable disease  Progressive disease | 1 (1.1)  19 (21.1)  33 (36.7)  28 (31.1) |

Data are presented as median (range) or n (%). ECOG PS, Eastern Cooperative Oncology Group performance status; ADC, adenocarcinoma; SqCC, squamous cell carcinoma; LN, lymph node; ICI, immune checkpoint inhibitor

^a^Adenosquamous carcinoma and sarcomatoid carcinoma

^b^Atezolizumab, durvalumab

^c^Nivolumab, Pembrolizumab

^*^Some cases had missing values.

**Supplementary Table S5.** Genes related to epithelial−mesenchymal transition, cytotoxic T-lymphocyte response, and regulatory T cell, M1, and M2 signature

| **Signature** | **Reference** | **Gene list** |
| --- | --- | --- |
| EMT  (for tumor) | Tan TZ et al. (2014) *EMBO Mol Med* | **Epi**: *KRT19, AGR2, RAB25, CDH1, ERBB3, FXYD3, SLC44A4, S100P, SCNN1A, GALNT3, PRSS8, ELF3, CEACAM6, TMPRSS4, CLDN7, TACSTD2, CLDN3, EPCAM, SPINT1, TSPAN1, PLS1, TMEM30B, PRR15L, KRT8, ST14, RBM47, S100A14, C1orf106, NQO1, TOX3, PTK6, TFF1, CLDN4, GPRC5A, TJP3, KRT18, MAP7, CKMT1A, ESRP1, MUC1, SPINT2, ESRP2, CDS1, PPAP2C, CEACAM7, TTC39A, OVOL2, EHF, AP1M2, CEACAM5, LAD1, ARHGAP8, TFF3, JUP, CD24, TMC5, MLPH, ELMO3, ERBB2, LLGL2, DDR1, FA2H, CBLC, TMPRSS2, LSR, PERP, POF1B, MYO5C, RAB11FIP1, MAPK13, KRT7, CEACAM1, CXADR, ATP2C2, RNF128, MPZL2, EPS8L1, GALNT7, CORO2A, BCAS1, TPD52, ARHGAP32, FUT2, OR7E14P, GALE, GRHL2, BIK, RAPGEFL1, STYK1, F11R, PKP3, CYB561, SH3YL1, GDF15, PSCA, EZR, TJP2, FGFR3, FUT3, BSPRY, TOM1L1, IRF6, EPB41L4B, SPDEF, OCLN, LRRC1, C19orf21, ABHD11, EPS8L2, MYO6, TSPAN8, MST1R, SLC16A5, GPR56, AZGP1, TOB1, SLC35A3, TRPM4, PHLDA2, VAMP8, SLC22A18, AKR1B10, VAV3, SPAG1, ABCC3, SYNGR2, STAP2, C4orf19, PPL, PLLP, DSG2, HDHD3, CD2AP, MANSC1, DHCR24, EPN3, TUFT1, GMDS, EXPH5, DSP, SDC4, IL20RA, FAM174B, PTPRF, SORD*  **Mes**: *GAS1, CXCL12, ZEB1, GLYR1, FHL1, FERMT2, C1S, FYN, WIPF1, CYP1B1, SERPING1, SERPINF1, VCAM1, MAP1B, TCF4, SRPX, EMP3, DPT, CALD1, PTGIS, VIM, CD163, C1R, FBN1, FN1, FXYD6, IGF1, NAP1L3, MRC1, QKI, MS4A4A, DCN, LOX, RECK, ANK2, LY96, ZFPM2, CSRP2, EFEMP1, RARRES2, PTPRC, PLEKHO1, RGS2, F13A1, JAM2, CHRDL1, TUBA1A, AP1S2, MYLK, DDR2, DSE, SACS, GLIPR1, CXCL13, FLRT2, PTX3, AKT3, COL6A2, DPYSL3, CDH11, PDZRN3, ZEB2, CCL2, MAFB, SFRP1, C14orf139, MFAP4, MAF, UCHL1, TUBB6, SRGN, HEG1, KCNJ8, AKAP12, EVI2A, COL14A1, AXL, ECM2, FSTL1, PLN, MYL9, OLFML3, STON1, SLIT2, BICC1, SOBP, CLIC4, ENPP2, SAMSN1, TPM2, ASPN, COL6A1, IGFBP5, MOXD1, AKAP2, SLC2A3, OLFML2B, ANGPTL2, PCOLCE, COLEC12, CTSK, TAGLN, CDH2, IL10RA, C1orf54, CEP170, TNS1, CLEC2B,* *JAM3, SEPT6, GREM1, VCAN, ZCCHC24, CRYAB, CFRP4, RUNX1T1, FGL2, MS4A5A, PTRF, GIMAP4, TWIST1, GFPT2, LHFP, CXCR4, SPOCK1, SPARC, VSIG4, GPM6B, TRPC1, SNAI2, GUCY1B3, PLXNC1, SYT11, FLI1, MYH10, CSF2RB, TNC, PMP22, COL5A2, MMP2, GNG11, CAV1, CDK14, SDC2, PTGDS, NR3C1, SYNM, FAP, NUAK1, WWTR1, FBLN1, MPDZ, SYNE1, EFEMP2, GIMAP6, KIAA1462, CCL8, COL15A1, CHN1, CRISPLD2, PDGFC, GEM, ISLR, GZMK, SPARCL1, BNC2, BGN, MEOX2, ITM2A, IFFO1* |
| EMT  (for cell line) | Tan TZ et al. (2014) *EMBO Mol Med* | **Epi**: *CDH1, AGR2, EPCAM, KRT19, RAB25, TACSTD2, S100P, CEACAM6, GALNT3, FXYD3, SPINT2, TMEM30B, SCNN1A, ST14, ESRP1, S100A14, CLDN7, ERBB3, RBM47, SPINT1, ELF3, CLDN4, PRSS8, SH3YL1, EHF, LCN2, JUP, VAMP8, KRT8, C1orf106, KRT7, DSP, CDS1, ITGB4, TMPRSS4, LSR, SORL1, GRHL2, PPL, C1orf116, TSPAN1, MAP7, SLPI, TOX3, ARHGAP8, F11R, LAD1, GPX2, CTSH, GPR56, FA2H, KLF5, AREG, KRT18, SCEL, CDH3, UGT1A1, MPZL2, AIM1, OVOL2, LLGL2, ESRP2, MYO5C, DDR1, VGLL1, IRF6, SFN, TSPAN13, KCNK1, MYO1D, PKP3, ITGB6, LY75, MAPK13, TTC39A, ELMO3, CEACAM1, DTX4, ERBB2, RAB11FIP1, ATP2C2, MST1R, AP1M2, TGFA, MYO6, PTK6, OAS1, FBP1, AQP3, CBLC, EPHA1, BSPRY, SH2D3A, EPS8L1, GRB7, C4orf19, KLK6, TJP2, PLS1, DENND2D, EPS8L2, IL20RA, HES1, IL1RN, EXPH5, ARHGDIB, C19orf21, CAMK2N1, HPGD, SYNGR2, C10orf116, PERP, MANSC1, DSC2, POF1B, SERINC5, BIK, ANXA9, MALL, EPN3, STAP2, FOXA1, PYCARD, ZNF165, SLC37A1, ANK3, TSPAN15, HNMT, ABCC3, SDC1, CKMT1A, TOB1, B3GNT3, TMC6, CD9, ADAP1, ATP1B1, SHANK2, SYB561, ERMP1, RAB20, MYH14, CAPN1, ALDH3B2, TRIM31, ARAP2, SSH3, ICA1, ARHGEF5, ALOX5, RHOD, TMPRSS2, MTUS1, CYP4F3, PPFIBP2, RABGAP1L, PLXNB2, MGST2, OR7E14P, EVPL, CD46, KRT15, CNKSR1, BLNK, COMT, ANXA4, TNFSF13, OCLN, SLC9A3R1, XBP1*  **Mes**: *VIM, ZEB1, EMP3, SACS, AXL, LOXL2, SPARC, FHL1, FERMT2, TUBA1A, TMEM158, CALD1, LGALS1, PMP22, MSN, GLYR1, MAP1B, AP1S2, GJA1, DENND5A, C12orf24, TPM2, TUBB6, SRPX, ANK2, CHN1, SH2B3, LEPRE1, ETV1, SOBP, AKAP12, TGFB1I1, SERPINE1, SOAT1, COL5A2, LHFP, CEP170, POPDC3, TRPC1, KDELC1, MYL9, BAG2, FSTL1, MXRA7, GFPT2, RECK, TMEFF1, PTRF* |
| CTL  response | Jiang P et al. (2018) *Nat. Med.* | *CD8A, CD8B, GZMA, GZMB, PRF1* |
| Treg | Andreatta M et al. (2021) *Nat Commun* | *TNFRSF4, TNFRSF1B, TNFRSF18, IL2RA, CTLA4, TIGIT, ICOS, GPX1, MAF, FOXP3, BATF, IKZF4, IKZF2, SAT1, TBC1D4, DUSP4, DNPH1, PHLDA1, SNX9, CD4, TYMP, NAMPT, SYNGR2, PBXIP1, GLRX* |
| M1 | Zhang L et al. (2020) *Cell*, and Azizi E et al. (2018) *Cell* | *CCL5, CCR7, CD40, CD86, CXCL9, CXCL10, CXCL11, IDO1, IL1A, IL1B, IL6, IRF1, IRF5, KYNU* |
| M2 | Zhang L et al. (2020) *Cell*, and Azizi E et al. (2018) *Cell* | *CCL4, CCL13, CCL18, CCL20, CCL22, CD276, CLEC7A, CTSA, CTSB, CTSC, CTSD, FN1, IL4R, IRF4, LYVE1, MMP9, MMP14, MMP19, MSR1, TGFB1, TGFB2, TGFB3, TNFSF8, TNFSF12, VEGFA, VEGFB,* and *VEGFC* |

Epi, epithelial; Mes, mesenchymal.

**Supplementary Table S6.** Primer sequences for deletion mutagenesis and cloning.

| **Target gene** | **Species** | **Strand** | **Sequence (5′→3′)** |
| --- | --- | --- | --- |
| PD-L1 ECD-TMD deletion | Human | Forward | CGT TTA AGA AAA GGG AGA ATG ATG GAT GTG AAA |
|  |  | Reverse | CCC TTT TCT TAA ACG CAT GTC GAC AAG GGC GAA |
| PD-L1 TMD-ICD deletion | Human | Forward | TAA GCG GCC GCA TCG TGA CTG ACT GAC GAT CTG |
|  |  | Reverse | CGA TGC GGC CGC TTA CCT TTC ATT TGG AGG ATG |
| TGFβ promoter insert (ATF2/c-Jun-binding motif) | Human | Forward | GGTACC (KpnI) gtg ctg agg gac tct gcc tcc aac gtc acc acc atc cac acc ccg gac acc cag tga tgg ggg agg atg gca cag tgg tca aga gca cag act cta gag act gtc aga gct gac ccc agc taa |
|  |  | Reverse | CGATCG (NheI) tta gct ggg gtc agc tct gac agt ctc tag agt ctg tgc tct tga cca ctg tgc cat cct ccc cca tca ctg ggt gtc cgg ggt gtg gat ggt ggt gac gtt gga ggc aga gtc cct cag cac |

**Supplemental References**

1. Tan TZ, Miow QH, Miki Y, Noda T, Mori S, Huang RY et al. Epithelial-mesenchymal transition spectrum quantification and its efficacy in deciphering survival and drug responses of cancer patients. EMBO Mol Med 2014;6:1279-1293.
2. Jiang P, Gu S, Pan D, Fu J, Sahu A, Hu X et al. Signatures of T cell dysfunction and exclusion predict cancer immunotherapy response. Nat Med 2018;24:1550-1558.
3. Andreatta M, Corria-Osorio J, Müller S, Cubas R, Coukos G, and Carmona SJ et al. Interpretation of T cell states from single-cell transcriptomics data using reference atlas. Nat Commun 2021;12:2965.
4. Zhang L, Li Z, Skrzypczynska, Fang Q, Zhang W, O’Brien SA et al. Single-Cell Analyses Inform Mechanisms of Myeloid-Targeted Therapies in Colon Cancer. Cell 2020;181(2):442-459.
5. Azizi E, Carr AJ, Plitas G, Cornish AE, Konopacki C, Prabhakaran S et al. Single-Cell Map of Diverse Immune Phenotypes in the Breast Tumor Microenvironment. Cell 2018;174(5):1293-1308.
